# Physiological and comparative proteomic characterization of *Desulfolithobacter dissulfuricans* gen. nov., sp. nov., a novel mesophilic, sulfur-disproportionating chemolithoautotroph from a deep-sea hydrothermal vent

**DOI:** 10.3389/fmicb.2022.1042116

**Published:** 2022-12-01

**Authors:** Yurina Hashimoto, Shigeru Shimamura, Akihiro Tame, Shigeki Sawayama, Junichi Miyazaki, Ken Takai, Satoshi Nakagawa

**Affiliations:** ^1^Laboratory of Marine Environmental Microbiology, Division of Applied Biosciences, Graduate School of Agriculture, Kyoto University, Kyoto, Japan; ^2^Institute for Extra-cutting-edge Science and Technology Avant-garde Research (X-star), Japan Agency for Marine-Earth Science and Technology (JAMSTEC), Yokosuka, Japan; ^3^General Affairs Department, Japan Agency for Marine-Earth Science and Technology (JAMSTEC), Yokosuka, Japan; ^4^Department of Marine and Earth Sciences, Marine Works Japan Ltd., Yokosuka, Japan; ^5^Section for Exploration of Life in Extreme Environments, Exploratory Research Center on Life and Living Systems (ExCELLS), National Institute of Natural Sciences, Okazaki, Japan

**Keywords:** sulfur disproportionation, *Deltaproteobacteria*, *Desulfobulbaceae*, deep-sea hydrothermal vent, comparative proteomic analysis, chemolithoautotroph

## Abstract

In deep-sea hydrothermal environments, inorganic sulfur compounds are important energy substrates for sulfur-oxidizing, -reducing, and -disproportionating microorganisms. Among these, sulfur-disproportionating bacteria have been poorly understood in terms of ecophysiology and phylogenetic diversity. Here, we isolated and characterized a novel mesophilic, strictly chemolithoautotrophic, diazotrophic sulfur-disproportionating bacterium, designated strain GF1^T^, from a deep-sea hydrothermal vent chimney at the Suiyo Seamount in the Izu-Bonin Arc, Japan. Strain GF1^T^ disproportionated elemental sulfur, thiosulfate, and tetrathionate in the presence of ferrihydrite. The isolate also grew by respiratory hydrogen oxidation coupled to sulfate reduction. Phylogenetic and physiological analyses support that strain GF1^T^ represents the type strain of a new genus and species in the family *Desulfobulbaceae*, for which the name *Desulfolithobacter dissulfuricans* gen. nov. sp. nov. is proposed. Proteomic analysis revealed that proteins related to tetrathionate reductase were specifically and abundantly produced when grown *via* thiosulfate disproportionation. In addition, several proteins possibly involved in thiosulfate disproportionation, including those encoded by the YTD gene cluster, were also found. The overall findings pointed to a possible diversity of sulfur-disproportionating bacteria in hydrothermal systems and provided a refined picture of microbial sulfur disproportionation.

## Introduction

Deep-sea hydrothermal environments are extreme habitats characterized by dynamic physicochemical conditions formed by the mixing of reducing hydrothermal fluid and oxidizing seawater (Kelley et al., 2002), and host a diversity of microorganisms that use chemical energy obtained from inorganic redox substances (Nakamura and Takai, 2014; Dick, 2019; Zeng et al., 2021). Abundant inorganic sulfur compounds, in particular, serve as both electron donors and acceptors for various microbial populations such as sulfur-oxidizers and -reducers (Elsgaard et al., 1995; Nakagawa et al., 2004; Nakagawa and Takai, 2008; Sievert et al., 2008). In addition, the occurrence of sulfur-disproportionating bacteria has been recently recognized in deep-sea hydrothermal systems (Slobodkin et al., 2012, 2013; Slobodkina et al., 2017). Sulfur disproportionation is a microbial dissimilatory metabolism in which a single intermediately oxidized inorganic sulfur compound, i.e., elemental sulfur (S^0^; Equation 1), sulfite, thiosulfate (Equation 2), or tetrathionate (Equation 3), is simultaneously reduced and oxidized, resulting in the production of sulfide and sulfate (Bak and Cypionka, 1987; Bak and Pfennig, 1987). This process significantly contributes to the global biogeochemical sulfur cycle (Fossing and Jørgensen, 1990; Jørgensen, 1990).


(1) $${\mathrm{4S}}^{0}+{\mathrm{4H}}_{2}O\to {\mathrm{SO}}_{4}{\;}^{2-}+{\mathrm{3HS}}^{-}+{\mathrm{5H}}^{+}$$

(2) $$S_{2}O_{3}{\;}^{2-}+H_{2}O\to {\mathrm{SO}}_{4}{\;}^{2-}+{\mathrm{HS}}^{-}+H^{+}$$

(3) $${\mathrm{4S}}_{4}O_{6}{\;}^{2-}+{\mathrm{4H}}_{2}O\to {\mathrm{6S}}_{2}O_{3}{\;}^{2-}+S_{3}O_{6}{\;}^{2-}+{\mathrm{SO}}_{4}{\;}^{2-}+{\mathrm{8H}}^{+}$$

Sulfur disproportionation was firstly discovered in *Desulfovibrio sulfodismutans* isolated from anoxic sludge (Bak and Cypionka, 1987; Bak and Pfennig, 1987). To date, sulfur-disproportionating bacteria have been found in limited members of the phyla *Firmicutes*, *Thermodesulfobacteria*, *Nitrospirae*, and *Proteobacteria* (the classes *Deltaproteobacteria* and *Gammaproteobacteria* in particular; Finster, 2008; Slobodkin and Slobodkina, 2019; Umezawa et al., 2021). Many, but not all, sulfur-disproportionating bacteria are also capable of sulfate reduction as an alternative energy metabolism (Slobodkin and Slobodkina, 2019). Generally, their growth rates and yields under sulfur disproportionating conditions are much lower than those under sulfate-reducing conditions. Therefore, the ability of sulfur disproportionation is not thoroughly evaluated for previously characterized sulfate-reducing prokaryotes, and thus their diversity may be significantly underestimated.

Enzymes and intermediates involved in sulfur disproportionation are only partially understood. In deltaproteobacterial sulfur-disproportionating bacteria with an ability of sulfate reduction, sulfate-adenylyltransferase (Sat), adenylyl-sulfate reductase (Apr), and dissimilatory sulfite reductase (Dsr) likely play major roles in both sulfur disproportionation and sulfate reduction (Finster, 2008; Slobodkin and Slobodkina, 2019). Exceptionally, in S^0^-disproportionating *Desulfurella amilsii*, rhodanese-like sulfurtransferases have been hypothesized to play a key role instead of Sat, Apr, or Dsr (Florentino et al., 2019). In the first step of thiosulfate disproportionation by *Desulfovibrio sulfodismutans* and *Desulfocapsa sulfexigens*, thiosulfate reductase has been suggested to function in the cleavage of thiosulfate into sulfite and sulfide (Krämer and Cypionka, 1989; Frederiksen and Finster, 2003). To get a complete picture of the mechanism of sulfur disproportionation, it is important to know how commonly these known enzymes are used in diverse sulfur-disproportionating bacteria. In addition, the identification of unknown proteins is essential to fill in the missing pieces in this process.

Here, we report the isolation and characterization of the first mesophilic, chemolithoautotrophic, diazotrophic, and sulfur-disproportionating deltaproteobacterium from a deep-sea hydrothermal vent. We also aim to identify proteins involved in thiosulfate disproportionation pathway by a comparative proteomic analysis. This study represents the first proteomic study of sulfur-disproportionating *Desulfobulbaceae* species.

## Materials and methods

### Sample collection, enrichment, purification, and phylogenetic analyses

Sample collection and subsampling were performed as described previously (Hashimoto et al., 2021). Briefly, the chimney structure was obtained at the Suiyo Seamount, at a depth of 1,383 m (Supplementary Figure 1). The sample was used to inoculate MMJFE medium, which contained 1 g NaHCO_3_ and 20 mM ferrihydrite [prepared as described in Kostka and Nealson (1998)] per liter of modified MJ synthetic seawater under H_2_/CO_2_ (80:20; 300 kPa; Nakagawa and Takai, 2006). After 3 days of incubation at 35°C, the medium became turbid, and the color of ferrihydrite changed to black (probably due to iron sulfide). The dilution-to-extinction technique was used to obtain a pure culture (Baross and Deming, 1995). The purity was confirmed by microscopic observation, by repeated partial sequencing of the 16S rRNA gene, and by genome sequencing. The isolate was designated as strain GF1^T^ (=JCM 34117^T^ = DSM 111414^T^) and routinely cultured with MMJFE medium at 35°C unless otherwise noted.

The extraction of genomic DNA and determination and alignment of the sequence of the PCR product (1,490 bp) were performed as described previously (Hashimoto et al., 2021). A phylogenetic tree was constructed as mentioned in (Nagata et al., 2017). Briefly, the sequence of strain GF1^T^ was aligned with a subset of 16S rRNA gene sequences, and only unambiguously aligned nucleotide positions (1,214 bases) were used for phylogenetic analyses.

### Growth observation and chemotaxonomic analysis

Cells were observed using a light microscope (BX53, Olympus, Tokyo, Japan). Electron micrographs were captured using a transmission electron microscope (Tecnai G2 20; FEI, Hillsboro, OR) as described previously (Hashimoto et al., 2021).

The growth of the novel isolate was measured by direct cell counting after staining with 4′,6-diamidino-2-phenylindole (DAPI; Porter and Feig, 1980). Effects of NaCl concentration (15–45 g L^−1^) and pH on growth were determined at 35°C. The pH tested were 5.7, 6.1, 6.6, 6.8, and 7.4. The pH was stable during the cultivation period under sulfate-reducing condition.

Sulfide production was monitored colorimetrically using the methylene blue method with a PACKTEST (Kyoritsu Chemical-Check Lab, Tokyo, Japan). Sulfate concentration was measured by a high-pressure liquid chromatography system (Shimadzu, Kyoto, Japan) with a Shodex IC NI-424 column (Showa denko, Tokyo, Japan), and 8 mM p-hydroxybenzoic acid, 3.2 mM Bis-Tris, and 50 mM boric acid as the mobile phase at 40°C. Tetrathionate and thiosulfate were separated using Dionex IonPac AS25 column (Thermo Fisher Scientific) at 40°C and detected at 216 nm as described previously (Bak et al., 1993). The mobile phase consisted of 2.9 g L^−1^ NaCl dissolved in 70% acetonitrile and 10% methanol.

Disproportionation of S^0^ (1%, w/v), Na_2_SO_3_ (0.1%, w/v), Na_2_S_2_O_3_·5H_2_O (0.1%, w/v), or Na_2_S_4_O_6_·2H_2_O (0.1%, w/v) was tested in the sulfate-free MMJFE medium (prepared by substituting chloride salts for sulfate salts) under N_2_/CO_2_ (80:20; 300 kPa), with or without ferrihydrite (20 mM). Sulfur disproportionation was confirmed by the formation of sulfate and sulfide. When measuring changes in metabolite concentrations over time during sulfur disproportionation, 15 ml of medium was added to a 30 ml vial and 3 ml of cell cultures at the late-exponential growth phase was added from pre-culture.

To examine alternative electron donors and acceptors for autotrophic growth, each of the potential electron donors such as S^0^ (1%, w/v), Na_2_SO_3_ (0.1%, w/v), Na_2_S_2_O_3_·5H_2_O (0.1%, w/v) was examined in combination with S^0^ (1%, w/v), Na_2_SO_3_ (0.1%, w/v), Na_2_S_2_O_3_·5H_2_O (0.1%, w/v), NaNO_3_ (0.1%, w/v), ferric citrate (0.1%, w/v), 20 mM ferrihydrite, 20 mM hematite, or 20 mM goethite as the electron acceptors in the sulfate-free MMJFE medium without ferrihydrite under N_2_/CO_2_ (80:20; 300 kPa). In an attempt to test the growth on hydrogen gas as an electron donor, H_2_/CO_2_ (80:20) was used as the gas phase (300 kPa).

Heterotrophic growth of strain GF1^T^ was tested by adding each of the following substrates at 0.1% (w/v) to MMJFE medium without NaHCO_3_ under 100% H_2_ (300 kPa): glucose, fructose, galactose, xylose, ribose, mannose, rhamnose, maltose, lactose, cellobiose, sucrose, arabinose, melibiose, citrate, succinate, pyruvate, lactate, propionate, malate, butyrate, formate, acetate, fumarate, malonate, tartrate, ethanol, methanol, 2-propanol, butanol, glycerol, ethylene glycol, glycine, glutamate, taurine, and casamino acids. In addition, to assess the utilization of these organic compounds as an energy source, substrates were added to MMJFE medium under N_2_/CO_2_ (80:20; 300 kPa). Fermentative growth with these substrates was also determined using the sulfate-free MMJFE medium under N_2_/CO_2_ (80:20; 300 kPa).

The utilization of N_2_ as a nitrogen source was tested using MMJFE medium lacking all nitrogen sources under H_2_/CO_2_/N_2_ (40:10:50; 300 kPa). Nitrogen fixation under sulfur-disproportionating condition was also tested in the medium designed for sulfur disproportionation tests (see above) lacking all nitrogen sources under N_2_/CO_2_ (80:20; 300 kPa). Throughout the test, 1 ml trace element solution SL-10 (Widdel et al., 1983) was substituted for 10 ml trace mineral solution (Sako et al., 1996) in MMJFE medium (per liter) to remove all possible nitrogen sources.

To confirm nitrogen fixation by strain GF1^T^, ^15^N_2_ tracer assays were performed under hydrogenotrophic sulfate-reducing, and S^0^- or thiosulfate-disproportionating condition with 300 ml medium designed for nitrogen fixation test (see above). Immediately before inoculation, 20 ml of ^15^N_2_ (99.9 atom%, Shoko Science Co., Ltd., Kanagawa, Japan) was added to the headspace of a 1,100 ml glass bottle. Control experiments were performed by using ^15^N_2_-free medium with or without inoculation. After cultivation, cells of the late-exponential growth phase were collected by filtration through 25 mm GF/F filters (0.3 μm pore size) that were pre-combusted at 400°C for 4 h. Cells on the filter were rinsed with 0.1 M HCl and immediately stored at −80°C until analysis. The nitrogen isotope ratios were determined by isotope ratio mass spectrometer (IRMS) coupled to an elemental analyzer (Flash 2000-DELTA plus Advantage ConFlo III System; Thermo Fisher Scientific) at Shoko Science Co.

Cellular fatty acids, respiratory quinones, and polar lipids of strain GF1^T^ were extracted and analyzed as described previously (Hashimoto et al., 2021). Cells of the late-exponential growth phase in MMJFE medium at 35°C were used.

### Genome sequencing and analyses

The whole genome sequencing and the contamination and quality check of the genome were conducted as described in (Hashimoto et al., 2021). The functional annotation was performed with DFAST (Tanizawa et al., 2018) and RAST v2.0 (Aziz et al., 2008). Metabolic pathways were predicted using the Kyoto Encyclopedia of Genes and Genomes (KEGG) based on the list of functional genes, which were automatically annotated by DFAST and then manually curated with the BLAST algorithm (Altschul et al., 1997). The HydDB webtool was used for hydrogenase classification^1^ (Søndergaard et al., 2016). Protein subcellular localization was predicted using PSORTb v3.0.3 (Yu et al., 2010). Average nucleotide identity (ANI) and average amino acid identity (AAI) were calculated with OrthoANI (Lee et al., 2016) and the Kostas lab AAI calculator,^2^ respectively.

A maximum-likelihood phylogenomic tree was reconstructed based on a total of 145 concatenated single-copy marker genes (Supplementary Table 1) by using ezTree software with the default parameters: Jones-Taylor-Thornton (JTT) model and 1,000 bootstraps (Wu, 2018). The resulting tree was then visualized using MEGA X v10.2.6 (Kumar et al., 2018).

### Shotgun proteomics sample preparation and LC–MS/MS analysis

For the shotgun proteomics, cells cultured under hydrogenotrophic sulfate-reducing and thiosulfate-disproportionating conditions were harvested at the late-exponential growth phase. Cells (about 10^10^ cells) were suspended in 400 μl of 100 mM triethylammonium bicarbonate (TEAB; pH 8.6) containing 2 mM phenylmethylsulfonyl fluoride (PMSF) and disrupted by sonication using a Q700 sonicator at a frequency of 40 kHz (QSonica, Newtown, CT, United States). The protein concentration of the cell-free extract was determined by a Qubit fluorometer (Thermo Fisher Scientific). The remaining procedure and LC–MS/MS data acquisitions were carried out as described previously (Kawai et al., 2022).

### Proteomics analysis

Protein identification was performed with the Proteome Discoverer 2.2 software package (Thermo Fisher Scientific). The acquired spectra were searched against the list of CDSs identified in the genome of strain GF1^T^ using the SEQUEST HT search algorithm, as described previously (Kawai et al., 2022). The results from biological triplicates were combined and filtered with a cut-off value of 1% protein false discovery rate (FDR) calculated using Protein FDR Validator node. A final dataset was generated to contain only proteins present in all three replicates. Relative protein abundances were calculated using normalized spectral abundance factors (NSAFs) method based on the number of PSMs per protein (Zybailov et al., 2006). NSAFs were then multiplied by 100 to calculate the relative protein abundance (percentage). For comparative proteomics, proteins with an average relative protein abundance of at least 0.1% in two tested conditions were mainly included in order to avoid overestimation.

### Orthology analysis

Protein sequences derived from a total of 93 bacterial genomes were obtained from NCBI Datasets. Analyzed species belonged to the classes *Clostridia*, *Nitrospira*, *Deltaproteobacteria*, and *Thermodesulfobacteria*. These included 87 strains from a previous dataset used for comparative genomics of sulfur-disproportionating bacteria (Umezawa et al., 2020), five species newly added in this study *Dissulfurispira thermophila* T55J^T^ (Umezawa et al., 2021), *Desulfovibrio desulfuricans* DSM 642^T^ (Bak and Pfennig, 1987; Galushko and Kuever, 2019), *Dissulfurimicrobium hydrothermale* Sh68^T^ (Slobodkin et al., 2016; Yvenou et al., 2022), *Thermosulfurimonas marina* SU872^T^ (Frolova et al., 2018), and *Thermosulfuriphilus ammonigenes* ST65^T^ (Slobodkina et al., 2017), and strain GF1^T^. Of the 93 species, 33 were sulfur-disproportionating bacteria. Proteins were grouped into orthologues by using OrthoFinder v2.5.4 with BLAST as the sequence search program (Emms and Kelly, 2015; Emms and Kelly, 2019). The identified ortholog groups were manually annotated based on the protein description of strain GF1^T^ within each group. To identify thiosulfate reductase-type protein or tetrathionate reductase-type protein, proteins corresponding to thiosulfate reductase-type protein subunit A (PhsA) or tetrathionate reductase-type protein subunit A (TtrA) were manually selected based on ortholog groups and manual checking of the amino acid length. Then, other subunits were also manually determined by checking the arrangement of genes in the vicinity of *phsA* or *ttrA* in the genome. Typically, the following arrangements were assigned as genes encoding thiosulfate reductase-type protein or tetrathionate reductase-type protein, respectively: *phsAB(C)* or *ttrBCA*. The combination of *ttrA* with distantly encoded *ttrBC* was also assigned as a single tetrathionate reductase-type protein.

## Results

### Cell morphology and phylogeny

**Figure 1:**
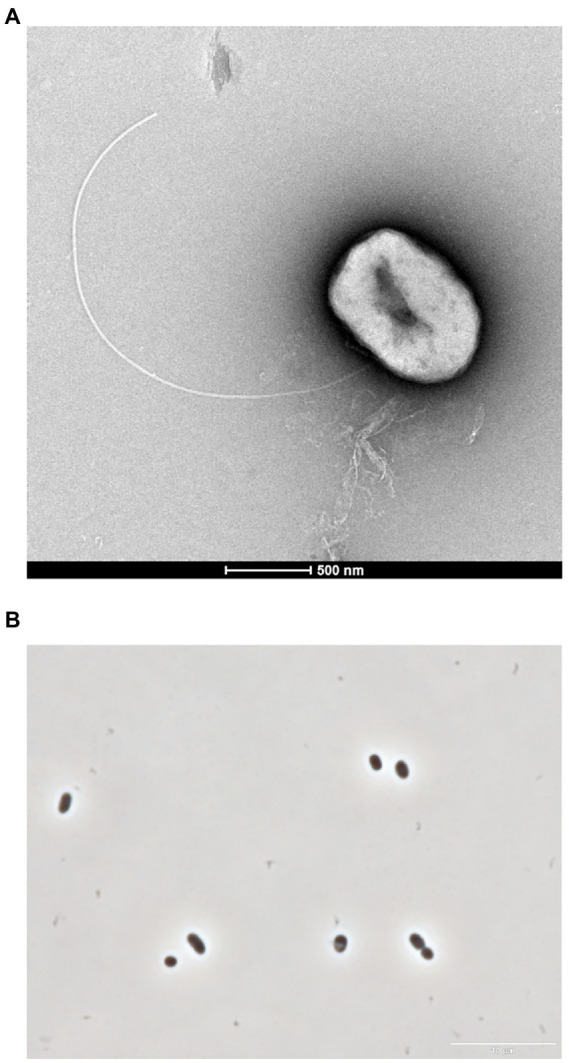
**(A)** Transmission electron micrograph showing a cell of GF1^T^. Scale bar represents 0.5 μm. **(B)** Phase contrast micrograph of strain GF1^T^. Scale bar represents 10 μm.

The cells of strain GF1^T^ were Gram-negative and motile short rods with a single flagellum (1.0–2.1 μm in length and 0.5–1.1 μm in width; Figure 1). Spore formation was not observed throughout the microscopic observation in this study.

**Figure 2:**
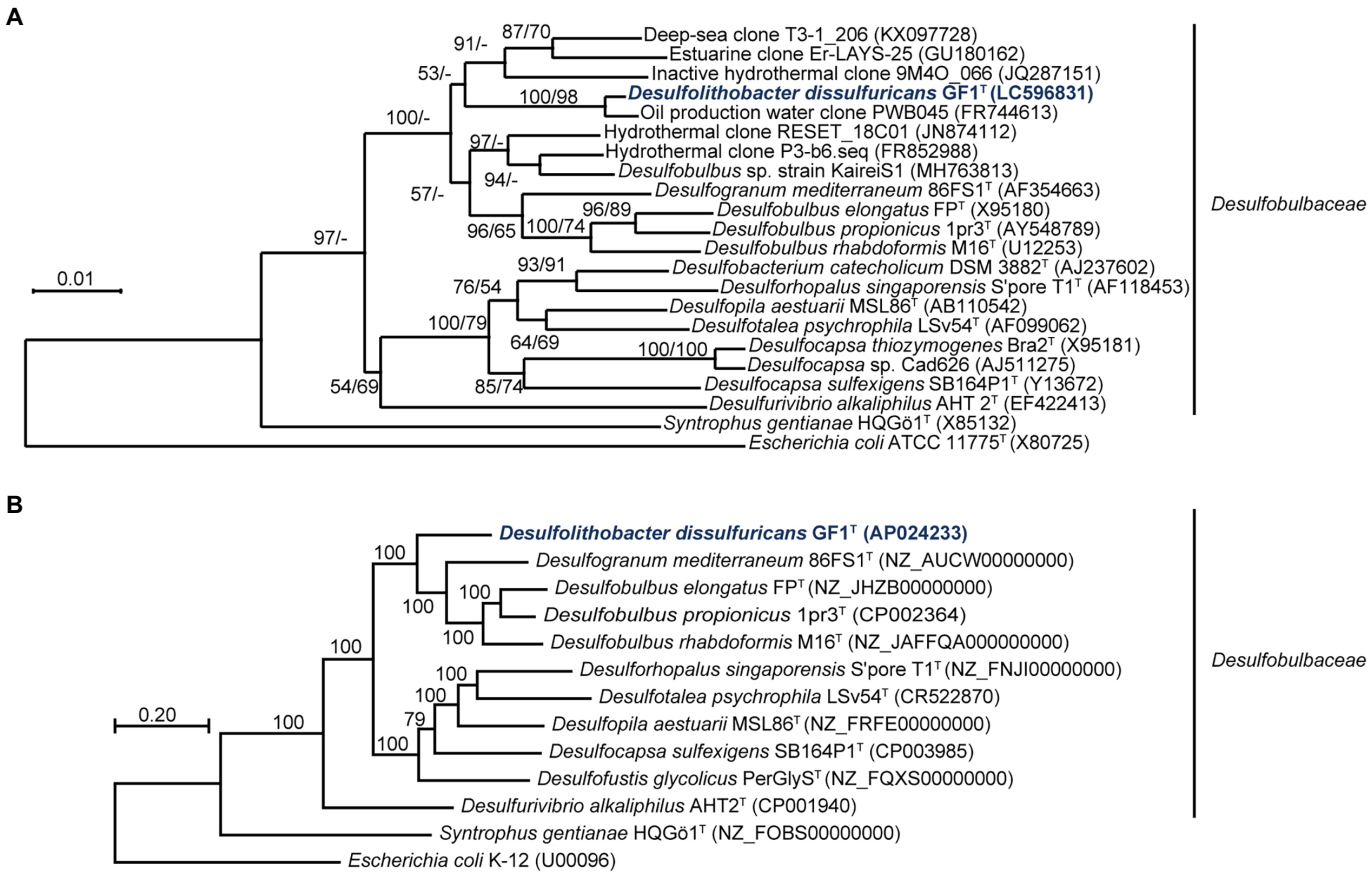
**(A)** Phylogenetic tree of representative members and environmental clones within the family *Desulfobulbaceae*, inferred from 16S rRNA gene sequences by the neighbor-joining method using 1,214 homologous sequence positions for each organism. Numbers at nodes are bootstrap values (percentages) based on 1,000 replicates (shown in the order neighbor-joining/maximum-likelihood). GenBank/EMBL/DDBJ accession numbers are given in parentheses. Bar, 0.01 changes per nucleotide position. **(B)** Maximum-likelihood phylogenetic tree of representative members within the family *Desulfobulbaceae*, based on concatenated 145 single-copy marker genes. Numbers at each node are bootstrap values (percentages) based on 1,000 replicates. GenBank/EMBL/DDBJ accession numbers are given in parentheses. Bar, 0.20 changes per nucleotide position.

The 16S rRNA gene sequence of strain GF1^T^ showed 98.2% similarity to that of an uncultured bacterium clone, PWB045, retrieved from the Halfdan oil field in the North Sea (Gittel et al., 2012). The closest cultured species were *Desulfogranum mediterraneum* 86FS1^T^ (93.3%; Sass et al., 2002) and *Desulfobulbus* sp. strain KaireiS1 (92.9%; Adam et al., 2021). These similarity values are below the common index of 16S rRNA gene sequence similarity for differentiation of microorganisms at the genus level (94.5%; Yarza et al., 2014; Figure 2A).

### Growth and chemotaxonomic characteristics

Strain GF1^T^ grew in the range of temperatures (25°C–50°C), NaCl concentrations (20–40 g L^−1^), and pH (6.1–6.8). The optimum growth occurred at 35°C, pH 6.6, and 3.0% (w/v) NaCl (Supplementary Figure 2). The generation time and maximum cell density under the optimal conditions were about 7.6 h and 2.2 × 10^7^ cells ml^−1^, respectively. During growth, the decrease in sulfate and the production of sulfide were observed (data not shown), confirming that strain GF1^T^ was a chemolithoautotrophic sulfate reducer.

Strain GF1^T^ did not grow on any of the tested organic compounds as a carbon source. However, the isolate was able to use formate or casamino acids as the sole energy source in the presence of CO_2_. Slow growth was observed when malate was provided as an electron donor (Table 1). No fermentative growth occurred with any tested substrates.

**Table 1:** Comparison of physiological characteristics of strain GF1^T^ with related genera of *Desulfobulbaceae*.

Characteristic	1	2	3	4	5	6
Temperature range (°C)	25–50	10–30	10–43	20–30	4–35	15–37
Temperature optimum (°C)	35	25	39	30	30	28
pH range	6.1–6.8	6.3–8.0	6.0–8.6	6.8–8.0	6.0–8.2	6.7–8.3
pH optimum	6.6	nr	7.1–7.5	7.3–7.5	6.7–7.3	7.3
NaCl range (%, w/v)	2.0–4.0	1.0–7.0	nr	nr	nr	nr
NaCl optimum (%, w/v)	3.0	2.0	nr	nr	nr	2.0
Electron donors:						
H_2_	+	−	+^†^	−	−	+^†^
Formate	+	−	−	−	−	−
Propionate	−	+	+	−	−	−
Pyruvate	−	+	+	−	−	nr
Lactate	−	+	+	−	−	+
Succinate	−	+	−	−	−	+
Fumarate	−	+	−	−	−	+
Malate	+	+	−	−	−	+
Electron acceptors:						
Sulfate	+	+	+	+	−	+
Sulfite	+	+	+	−	+	+
Thiosulfate	+	+	+	−	+	−
Nitrate	−	−	+	−	−	−
Disproportionation:						
S^0^	+	nr	+	+	+	+
Sulfite	−	−	−	+	+	nr
Thiosulfate	+	−	+	+	+	nr
Tetrathionate	+	nr	nr	nr	nr	nr
DNA G + C content (mol%)	56.9^a^	58.6	59.9	50.7	47.2 ± 0.2	56.2 ± 0.1
Major cellular fatty acids*	C_16:0_, C_18:1_, C_16:1_, C_18:0_	C_16:1_, C_18:1_, C_17:1_, C_14:0_	C_16:1_, C_17:1_, C_18:1_, C_14:0_	nr	nr	nr
Major quinone	MK-8^b^	nr	MK-5(H_2_)	nr	nr	MK-5(H_2_)

1, GF1^T^ (this study); 2, *Desulfogranum mediterraneum* 86FS1^T^ (Sass et al., 2002); 3, *Desulfobulbus propionicus* 1pr3^T^ (Widdel and Pfennig, 1982; Collins and Widdel, 1986; Krämer and Cypionka, 1989; Lovley and Phillips, 1994; Sass et al., 2002); 4, *Desulfocapsa thiozymogenes* Bra2^T^ (Janssen et al., 1996); 5, *Desulfocapsa sulfexigens* SB164P1^T^ (Finster et al., 1998; Frederiksen and Finster, 2004); 6, *Desulfofustis glycolicus* PerGlyS^T^ (Friedrich et al., 1996; Finster, 2008). +, positive; −, negative; nr, not reported.

a Value determined from genome sequence.

b The degree of saturation of menaquinone side chain was not determined.

* Cellular fatty acids were analyzed using cells grown in different cultivation conditions.

† Grew only in the presence of acetate.

**Figure 3:**
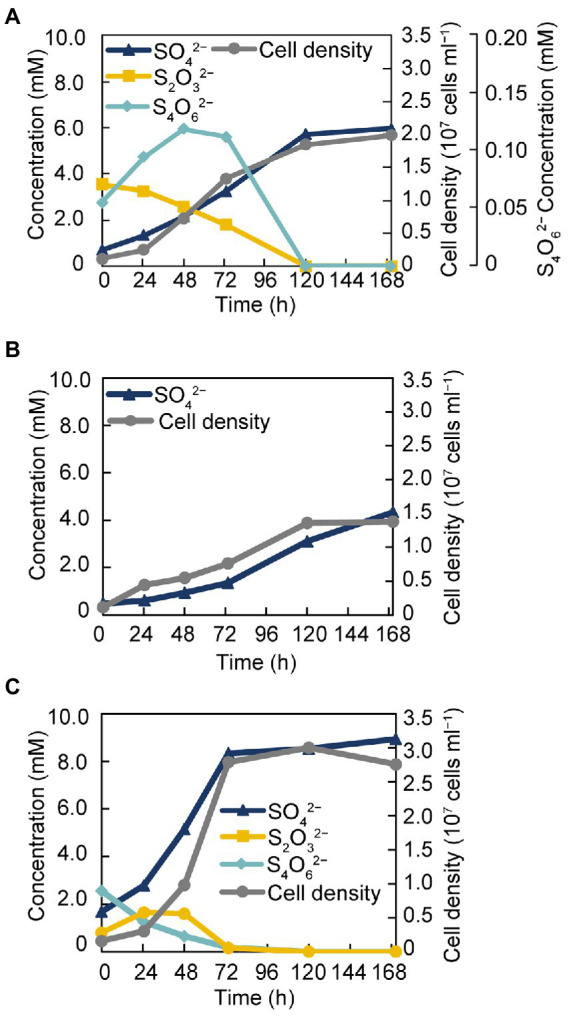
Growth of strain GF1^T^ *via* disproportionation of thiosulfate **(A)**, S^0^ **(B)**, and tetrathionate **(C)**.

The isolate was found to utilize H_2_ as an electron donor and sulfate, sulfite, and thiosulfate as electron acceptors (Table 1). In addition, strain GF1^T^ was able to grow *via* disproportionation of S^0^ (38 h doubling time), thiosulfate (20 h doubling time), and tetrathionate (17 h doubling time) only in the presence of ferrihydrite as a sulfide scavenger. The final cell yield under sulfur disproportionation was almost the same as that under sulfate-reducing condition (about 10^7^ cells ml^−1^). Although elemental sulfur could not be quantified, a decrease of thiosulfate/tetrathionate and a concomitant increase of sulfate and cell density was observed (Figure 3). During thiosulfate disproportionation, a trace amount of tetrathionate was produced and then consumed as the cell density increased (Figure 3A). In the abiotic control, no such change in tetrathionate concentration was detected. Similarly, slight thiosulfate production and subsequent consumption were observed during tetrathionate disproportionation (Figure 3C). Tetrathionate/thiosulfate present at hour 0 of incubation under thiosulfate/tetrathionate-disproportionating conditions were probably due to carryover from the pre-culture. Thiosulfate may also have been formed by the abiotic decomposition of tetrathionate in the presence of sulfide (Rowe et al., 2015). Sulfite did not serve as a substrate for disproportionation.

Diazotrophic growth was observed under both sulfate-reducing and sulfur-disproportionating conditions. N_2_ fixation was demonstrated by assimilation of ^15^N_2_ into cellular components (Supplementary Table 2).

The major cellular fatty acids of strain GF1^T^ were C_16:0_ (36.3%), C_18:1_ (26.5%), C_16:1_ (24.7%), and C_18:0_ (6.0%; Table 1). The respiratory quinone of strain GF1^T^ was menaquinone-8 (MK-8). Strain GF1^T^ possessed phosphatidylethanolamine (PE), phosphatidylglycerol (PG), and an unidentified polar lipid, as *Desulfogranum japonicum* Pro1^T^ did (Supplementary Figure 3).

### Genome characteristics

The genome of strain GF1^T^ was assembled into one circular chromosomal contig of 3,596,428 bases and contained 3,242 coding sequences (CDSs). The G + C content was 56.9%, and the mean depth of coverage was 292×. No genome contamination was confirmed by ContEst16S. The topology of the phylogenomic tree (Figure 2B) was generally consistent with that of the 16S rRNA gene-based phylogenetic tree (Figure 2A). The ANI and AAI between strain GF1^T^ and related genera of *Desulfobulbaceae* supported that the isolate could be differentiated at the genus level within the family *Desulfobulbaceae*, based on the previously proposed threshold (Goris et al., 2007; Richter and Rosselló-Móra, 2009; Konstantinidis et al., 2017; Supplementary Figure 4).

A complete gene set for dissimilatory sulfate reduction was found in the genome, including sulfate adenylyltransferase (*sat*), adenylylsulfate reductase subunit α/β (*aprAB*), dissimilatory sulfite reductase subunit α/β (*dsrAB*), *dsrC, dsrD*, and *dsrMKJOP* complex (Supplementary Table 3). Genes corresponding to APS reductase-associated electron transfer complex (*qmoABC*) were also identified. In addition, genome analysis revealed the presence of genes potentially involved in sulfur disproportionation, including molybdopterin oxidoreductases, which may act as a subunit of thiosulfate, polysulfide, or tetrathionate reductase.

In addition to Sat and Apr, sulfite oxidoreductase has also been proposed as a possible enzyme involved in the oxidative route of sulfur disproportionation (Finster, 2008), but no gene encoding sulfite oxidoreductase was identified in the genome of strain GF1^T^. Genes associated with the oxidation of S^0^ and thiosulfate, such as sulfur-oxidizing (sox) system, sulfide: quinone oxidoreductase (*sqr*), or sulfur oxygenase reductase (*sor*), were missing in the genome, as previously reported in several sulfur-disproportionating bacteria (Slobodkin and Slobodkina, 2019).

The genes of the Wood-Ljungdahl pathway for CO_2_ fixation were identified (Supplementary Table 3). For molecular hydrogen oxidization, the isolate harbored genes encoding membrane-anchored periplasmic [NiFe] hydrogenase (HynABC), cytoplasmic [NiFe] methyl-viologen-reducing hydrogenase, and hydrogenase accessory proteins. Genes from the TCA cycle were present in the genome of strain GF1^T^, which may be responsible for the metabolism of C_4_-dicarboxylates including malate. Further, all genes for C_4_-dicarboxylate transporters (Dct), which function in the uptake of C_4_ compounds, were detected in the genome (GF1_10880 to GF1_10900). Genes involved in amino acid transport were encoded in the genome. The genome also contained genes needed for nitrogen fixation, which was congruent with its ability to grow diazotrophically (Supplementary Table 3). Additionally, genes encoding flagella components were identified in the genome.

### Comparative proteomic analysis

**Figure 4:**
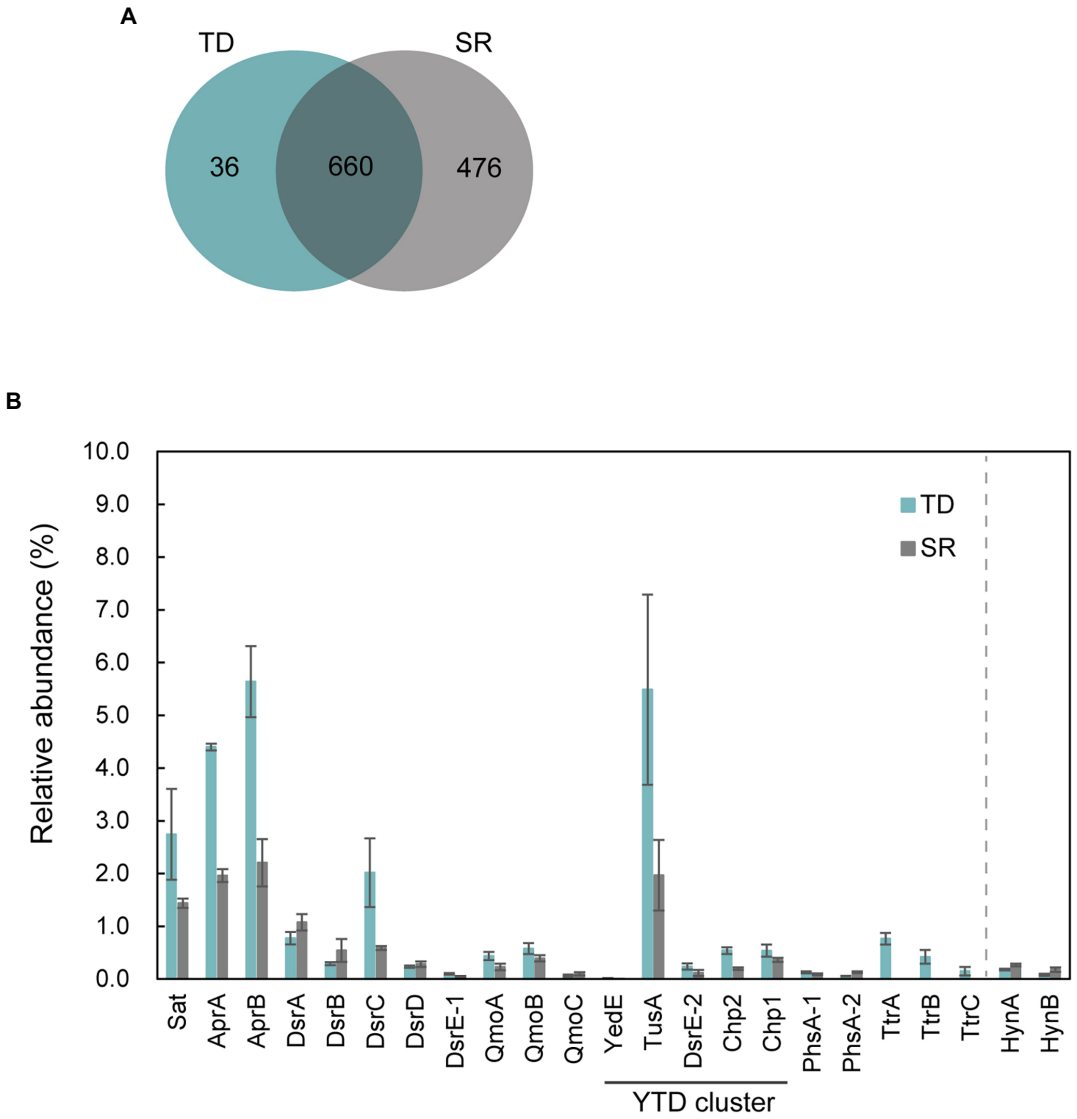
Proteomic profiles of strain GF1^T^ under thiosulfate disproportionation (TD) and sulfate reduction (SR). **(A)** Venn diagram of shared/unique proteins identified in the proteome of TD (Blue) and SR (Gray). **(B)** Relative abundance (%) of proteins potentially involved in sulfur metabolism found in the proteome of strain GF1^T^ under TD (Blue) and SR (Gray). Relative abundance is based on the average NSAF values of the biological triplicates. Error bars represent standard error of the biological triplicates. HynA/B is shown for comparison. The locus tag for each protein is listed in Supplementary Table 9.

To identify proteins that were specifically and/or abundantly produced when strain GF1^T^ grew *via* sulfur disproportionation, a comparative proteomic analysis was performed using cells grown under two different conditions: thiosulfate-disproportionating condition (hereinafter referred to as TD) and hydrogenotrophic sulfate-reducing condition (SR). We identified a total of 1,172 proteins (Figure 4A). Thereof, 36 and 476 were exclusively found in TD and SR, respectively. Of the 36 proteins exclusively found in TD, the top 10 most abundant proteins were listed in Table 2. The most abundant TD-specific protein exhibited a high level of amino acid sequence similarity (70.6%) to the molybdopterin-binding subunit A of tetrathionate reductase of *Dissulfuribacter thermophilus* (WP_067617696), that is a sulfur disproportionator originated from a deep-sea hydrothermal vent (Slobodkin et al., 2013). The other subunits of tetrathionate reductase-type protein, the electron transfer subunit B (TtrB) and the membrane-anchor subunit C (TtrC), were also identified only in TD (Table 2). In addition, a hypothetical protein encoded by a gene adjacent to *ttrBCA* in the GF1^T^ genome (GF1_03960) and another molybdopterin oxidoreductase (GF1_03980) were uniquely found in TD (Supplementary Table 4), suggesting its potential involvement in thiosulfate disproportionation. Other proteins exclusively found in TD contained subunits of NADH-quinone oxidoreductase (Nuo; GF1_30510, GF1_30520, GF1_31140, GF1_31220; Supplementary Table 4). In addition, a rhodanese-like domain-containing protein (Rhd; GF1_13980) was detected only in TD, which was in accordance with the proteome data from *Desulfurella amilsii* grown under S^0^-disproportionating condition (Florentino et al., 2019).

**Table 2:** Top 10 abundant proteins only detected in TD.

Protein description	BLAST top hit (Accession number)	Locus tag	Relative protein content (%)^a^	Rank^b^
Tetrathionate reductase-type protein subunit A (TtrA)	Tetrathionate reductase subunit TtrA (PLX49341)	GF1_03950	0.765	17
Tetrathionate reductase-type protein subunit B (TtrB)	Tetrathionate reductase subunit B (PLX49339)	GF1_03930	0.420	33
Universal stress protein	Universal stress protein family protein (GBE12839)	GF1_10800	0.173	105
Tetrathionate reductase-type protein subunit C (TtrC)	Polysulfide reductase NrfD (HFQ80664)	GF1_03940	0.149	127
Response regulator	Response regulator (HHD63890)	GF1_10760	0.115	172
Hypothetical protein	ABC transporter substrate-binding protein (HDO29561)	GF1_04120	0.103	190
tRNA-binding protein	tRNA-binding protein (MCF6341267)	GF1_29610	0.100	200
NADH-quinone oxidoreductase subunit B	NADH-quinone oxidoreductase subunit B (MCF6188143)	GF1_30520	0.084	244
Hypothetical protein	Universal stress protein (HEB49400)	GF1_10730	0.060	342
Hypothetical protein	Hypothetical protein (NOX26015)	GF1_03960	0.052	390
Thiosulfate reductase-type protein (PhsA-1)	Thiosulfate reductase (HEB50405)	GF1_30490	0.124	160
Thiosulfate reductase-type protein (PhsA-2)	Thiosulfate reductase (HFQ88534)	GF1_10120	0.056	362

Protein descriptions are based on automatic annotation with DFAST and manual curation using the BLAST algorithm. Thiosulfate reductase-type proteins are listed for comparison.

a Based on the average NSAF values of the biological triplicates.

b Rank of relative protein content in TD.

The top 20 most abundant proteins in TD and SR are shown in Supplementary Table 5, and all proteins found in TD and SR are listed in Supplementary Tables 6, 7, respectively. Among 660 proteins detected in both TD and SR, relatively abundant proteins in TD included proteins possibly involved in sulfur metabolism (Figure 4B, Supplementary Tables 8, 9); DsrC, the substrate for DsrAB in dissimilatory sulfate reduction pathway (Santos et al., 2015), was found to be 3.4-fold more abundantly produced in TD than in SR (GF1_24490). Sat and AprAB, which have been suggested to function in the oxidative route of sulfur disproportionation, were even more abundantly produced in TD (more than 1.5-fold change). APS reductase-associated electron transfer complex (QmoABC) was also abundantly produced in TD, except for transmembrane subunit QmoC. In contrast, DsrAB and its allosteric activator DsrD, which work in sulfate reduction and potentially in the reductive branch of sulfur disproportionation, were slightly less produced in TD than in SR. DsrE/F like family protein (hereafter DsrE-1; GF1_04100) was present in both conditions, although the relative protein content was <0.1%.

In addition, it was noteworthy that all five proteins (referred to as YedE, TusA, DsrE-2, Chp2, and Chp1) encoded by the YTD gene cluster (GF1_30690 to GF1_30730) were more abundant in TD (Figure 4B), although the relative protein content of YedE (GF1_30690) was <0.1% (0.01% in TD and 0.007% in SR). The YTD cluster is reported to potentially have an important role in microbial sulfur disproportionation, but its function has not been determined in detail (Umezawa et al., 2020).

Moreover, two proteins corresponding to thiosulfate reductase-type protein subunit A (PhsA), molybdopterin-containing oxidoreductase, were detected in both TD and SR. PhsA-1 (GF1_30490) was slightly more abundant in TD and PhsA-2 (GF1_10120) was more abundant in SR (Figure 4B). Another subunit of thiosulfate reductase-type protein, PhsB, was also found in both TD and SR (PhsB-1, GF1_30500 and PhsB-2, GF1_10130; Supplementary Tables 6, 7). Further, a full set of enzymes required for CO_2_ fixation by the Wood-Ljungdhal pathway were detected in both TD and SR.

The SR-specific proteins contained proteins encoded by a hydrogenase gene cluster, including membrane-anchored periplasmic [NiFe] hydrogenase, cytoplasmic [NiFe] methyl-viologen-reducing hydrogenase/heterodisulfide reductase (mvh/hdr), and hydrogenase accessory proteins (GF1_18040, GF1_18060, GF1_18080, GF1_18140 to GF1_18220, GF1_18260 to GF1_18290; Supplementary Table 10). For membrane-anchored periplasmic [NiFe] hydrogenase (HynABC), only HynC (GF1_18080) was specifically present in SR, and HynAB (GF1_18090, GF1_18100) was more abundantly produced in SR than in TD (Figure 4B). The SR-specific most abundant protein (hypothetical protein encoded by GF1_18120) was also encoded by a gene within this hydrogenase cluster (Supplementary Figure 5), but its functional assignment was unclear. SulP family inorganic anion transporter (GF1_18110), encoded by a gene adjacent to GF1_18120, was also uniquely produced in SR.

### Orthologous proteins in diverse bacteria

Orthologs of the subunits of tetrathionate reductase-type protein in strain GF1^T^ were present in *Desulfitobacterium hafniense*, 12 other *Deltaproteobacteria*, and four *Thermodesulfobacteria* members (Supplementary Tables 11, 12). Of these, 11 species had complete TtrBCA. *Desulfoluna spongiiphila, Desulfosarcina cetonica*, *Desulfobulbus propionicus*, and *Caldimicrobium thiodismutans* had TtrAB cluster lacking TtrC. *Desulfuromusa kysingii* and *Dissulfuribacter thermophilus* had complete TtrBCA as well as TtrAB lacking TtrC. Of all these species, six deltaproteobacterial species and four thermodesulfobacterial species were reported to be sulfur-disproportionating bacteria. All of them were capable of thiosulfate disproportionation, except *Desulfurella amilsii*, which has not been tested for thiosulfate disproportionation. Of these sulfur-disproportionating species with orthologs of tetrathionate reductase-type protein, *Dissulfuribacter thermophilus*, *Dissulfurimicrobium hydrothermale*, and four thermodesulfobacterial species originated from thermal ecosystems. Further, all these six species had orthologous proteins encoded by the YTD gene cluster (Supplementary Table 13). *Thermosulfurimonas dismutans* and *Thermosulfurimonas marina* were the only thiosulfate-disproportionating species with an ortholog of tetrathionate reductase-type protein but lacking thiosulfate reductase-type protein. No other species with orthologs of tetrathionate reductase-type protein has reports on the ability of sulfur disproportionation, except *Desulfatirhabdium butyrativorans*, which is unable to disproportionate thiosulfate and sulfite (Balk et al., 2008). Ten thiosulfate-disproportionating species contained neither an ortholog of thiosulfate reductase-type protein nor an ortholog of tetrathionate reductase-type protein.

Orthologs of thiosulfate reductase-type protein in strain GF1^T^ were found in three *Clostridia*, four *Nitrospira*, 30 other *Deltaproteobacteria*, and nine *Thermodesulfobacteria* members (Supplementary Tables 11, 14). Among these species, 15 were identified as sulfur-disproportionating bacteria, eight were unable to disproportionate inorganic sulfur compounds, and 23 species have no reports on sulfur disproportionation. Sulfur-disproportionating species with orthologs of thiosulfate reductase-type protein included *Desulfotomaculum nigrificans*, 12 other deltaproteobacterial species, and two thermodesulfobacterial species. All these species except *Desulforhopalus singaporensis*, *Desulfovibrio cuneatus*, and *Paucidesulfovibrio longus* were able to disproportionate thiosulfate. *Desulfotomaculum nigrificans*, *Desulfobacter curvatus*, *Desulfocapsa sulfexigens*, *Desulfonatronospira thiodismutans*, and *Desulfovibrio aminophilus* were thiosulfate-disproportionating species with an ortholog of thiosulfate reductase-type protein but lacking tetrathionate reductase-type protein.

## Discussion

In this study, we isolated and characterized strain GF1^T^, which is the first mesophilic sulfur disproportionator from a deep-sea hydrothermal vent. Strain GF1^T^ differs from other sulfur-disproportionating bacteria from deep-sea hydrothermal systems in that it is a facultative sulfate reducer (Slobodkin et al., 2012, 2013; Slobodkina et al., 2017). In addition, strain GF1^T^ is unique in that it can utilize N_2_ as the sole nitrogen source under sulfur-disproportionating conditions and it is in line with the identification of *nifDK* genes in the genome. Although N_2_ fixation ability has not been well investigated in sulfur-disproportionating bacteria, *nif* genes have been found in the genomes of sulfur disproportionators, i.e., *Desulfocapsa sulfexigens*, *Thermosulfurimonas dismutans*, and *Dissulfurimicrobium hydrothermale* (Finster et al., 2013; Mardanov et al., 2016; Yvenou et al., 2022). Phylogenetic analysis showed that the isolate is closely related to the genus *Desulfogranum* but within a previously uncultivated cluster of *Desulfobulbaceae* members (Figure 2). Major cellular fatty acids of strain GF1^T^ are similar to that of the members of the genus *Desulfogranum* (Galushko and Kuever, 2020), whereas the strain exhibits a much higher percentage of C_16:0_. Further, the respiratory quinone of the isolate (MK-8) is different from the major quinone of the closely related genus *Desulfogranum* (MK-5(H_2_); Table 1; Galushko and Kuever, 2020). Based on the molecular, physiological, and chemotaxonomic analyses, we propose that strain GF1^T^ represents the type strain of a novel species in a new genus, *Desulfolithobacter dissulfuricans* gen. nov., sp. nov.

**Figure 5:**
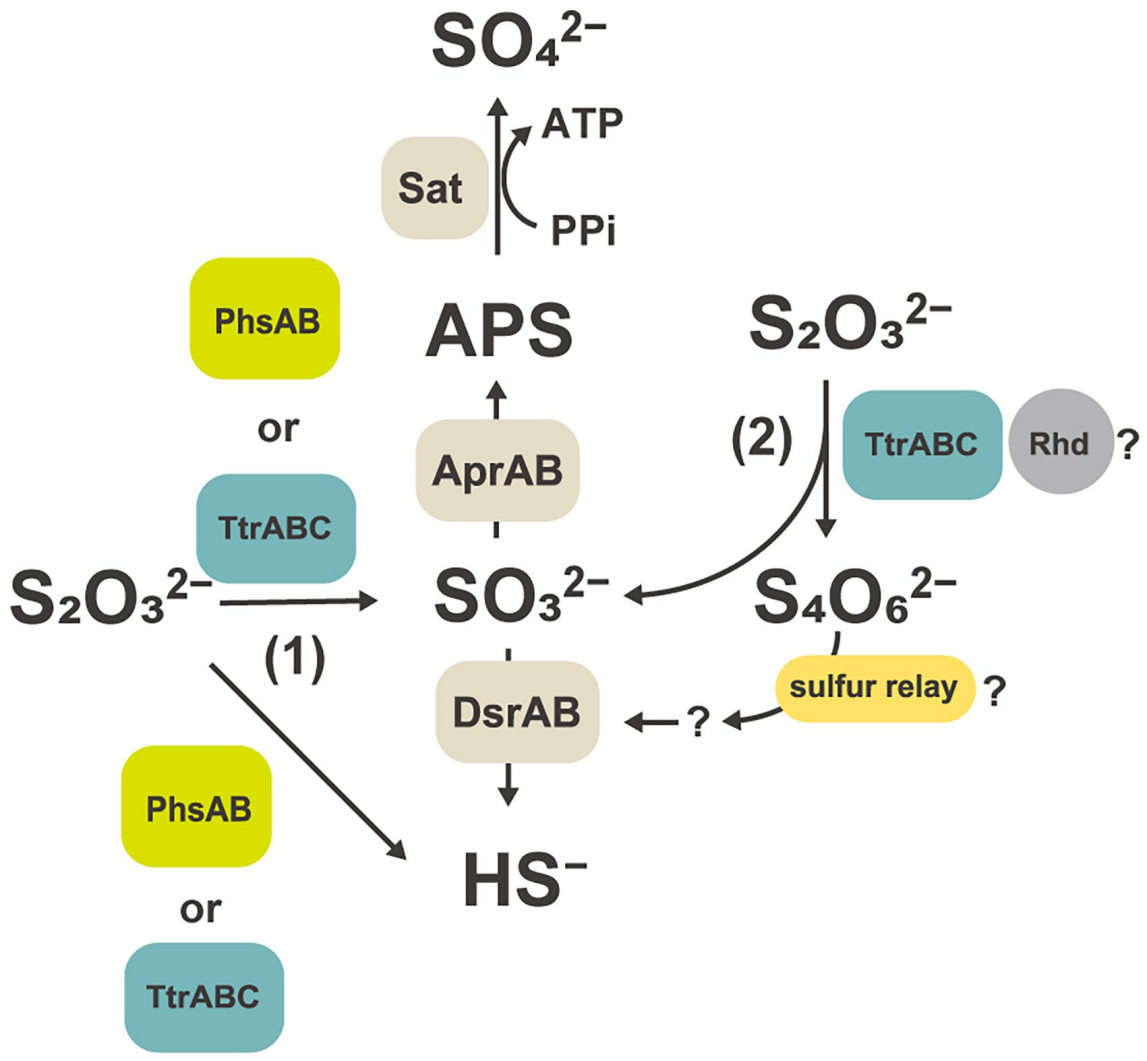
Hypothetical thiosulfate disproportionation pathways in strain GF1^T^ based on a comparative proteomic analysis. Sat, sulfate-adenylyltransferase; Apr, adenylyl-sulfate reductase; APS, adenosine 5′-phosphosulfate; Dsr, dissimilatory sulfite reductase subunit; Phs, thiosulfate reductase-type protein; Ttr, tetrathionate reductase-type protein; Rhd, rhodanese-like domain-containing protein.

Comparative proteomic study revealed that proteins related to the subunits of molybdopterin-containing tetrathionate reductase-type protein (GF1_03930 to GF1_03950) were specifically and abundantly produced (Table 2, Figure 4B) when strain GF1^T^ was grown *via* thiosulfate disproportionation. Tetrathionate reductase is an enzyme that is responsible for the reduction of tetrathionate to thiosulfate (Hensel et al., 1999), but our result indicated its possible involvement in thiosulfate disproportionation by strain GF1^T^. Given that thiosulfate is simultaneously reduced and oxidized during disproportionation, it is possible that identified tetrathionate reductase-type protein could play a major role in either oxidation or reduction step (Figure 5). However, it should be noted that it is thermodynamically unfavorable to oxidize thiosulfate to tetrathionate (*E*_m_ = +180 mV) using the MK-8 (*E*_m_ = −70 mV) identified in our strain.

Tetrathionate reductase-type protein has been suggested to be functionally diverse; for example, sequences showing a BCA order of genes but not clustering with tetrathionate reductase-type protein were identified and whose metabolic function is still unclear (Duval et al., 2008). Further, tetrathionate reductase-type sequence which may function as dissimilatory As(V) reductases, lacking tetrathionate reductase activity, was reported in (Muramatsu et al., 2020). Therefore, further biochemical and enzymological characterization would be needed to elucidate the function of tetrathionate reductase-type protein identified in this study.

Another unexpected result of the proteomic analysis was that the protein content of thiosulfate reductase-type protein was relatively low under thiosulfate disproportionation condition (0.12%, PhsA-1; 0.06%, PhsA-2; Figure 4B). This observation may imply that thiosulfate reductase, which is believed to function in the first step of thiosulfate disproportionation pathway (Krämer and Cypionka, 1989; Frederiksen and Finster, 2003), may not be a major player in thiosulfate disproportionation in our isolate. In addition, Phs identified in this study lacked subunit C. To date, no functional analysis of PhsAB has been performed, and C subunit is considered to be essential for the enzyme to interact with its co-substrate, quinone. However, a recent study proposed that PhsAB in a deltaproteobacterial *Desulfurella amilsii* can mediate the cleavage of thiosulfate into sulfite and sulfide (Florentino et al., 2019).

**Figure 6:**
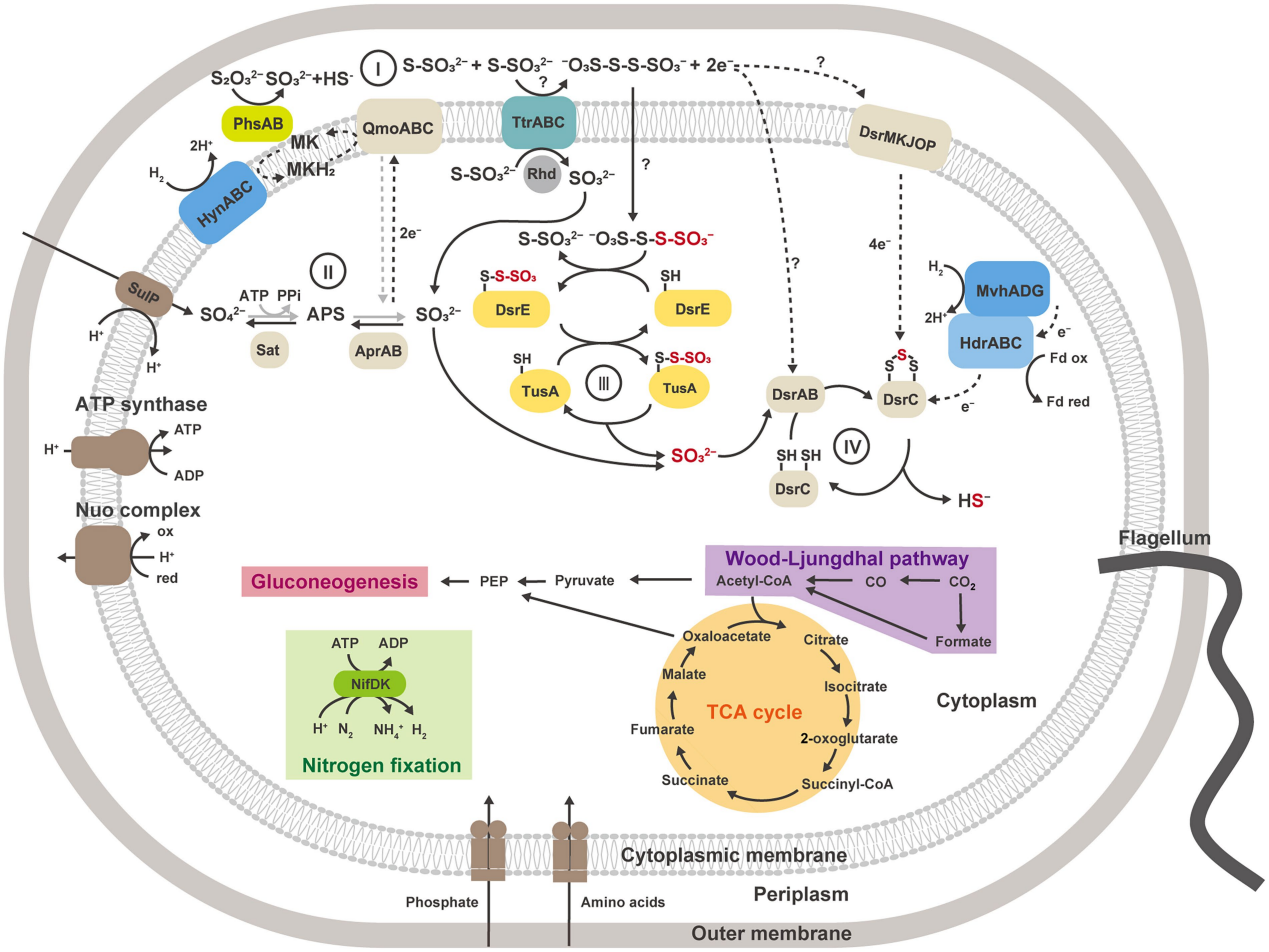
Metabolic reconstruction of strain GF1^T^ and proposed model for thiosulfate disproportionation in strain GF1^T^, involving the enzyme reaction by tetrathionate reductase-type protein (I), oxidation of sulfite to sulfate by Apr and Sat (II), possible transfer of thiosulfonate derived from tetrathionate by potential sulfur carrier proteins and delivery of sulfite to DsrAB (III), reduction of sulfite to sulfide by DsrAB (IV). The strain grows *via* sulfur disproportionation and respiratory hydrogen oxidation coupled to sulfate reduction in the presence of CO_2_. When reactions can work in both directions, the pathway for dissimilatory sulfate reduction is shown in gray. Sat, sulfate-adenylyltransferase; Apr, adenylyl-sulfate reductase; APS, adenosine 5′-phosphosulfate; Dsr, dissimilatory sulfite reductase subunit; Phs, thiosulfate reductase-type protein; Ttr, tetrathionate reductase-type protein; Rhd, rhodanese-like domain-containing protein; Qmo, APS reductase-associated electron transfer complex.

Moreover, in our proteomic study, all proteins encoded by the YTD gene cluster were more abundantly detected in thiosulfate-disproportionating condition (Figure 4B). This result supported the idea that the YTD gene cluster can be involved in sulfur disproportionation (Umezawa et al., 2020). Although its functional assignment is unclear, TusA and DsrE in the YTD gene cluster are known to be involved in sulfur transfer to the Dsr system in sulfur-oxidizing bacteria, using their conserved cysteine residues as sulfane sulfur binding sites (Stockdreher et al., 2012, 2014; Venceslau et al., 2014; Tanabe et al., 2019; Dahl, 2020). Taking into account that TusA (GF1_30700) and DsrE-2 (GF1_30710) encoded by the YTD gene cluster in strain GF1^T^ harbored conserved cysteine residues, these proteins can work as sulfur carrier proteins in strain GF1^T^. In addition, a recent study on DsrE/TusA homolog proteins from an acidothermophilic sulfur-oxidizing archaeon *Metallosphaera cuprina* has biochemically proven that tetrathionate reacts with DsrE/TusA homolog proteins and its thiosulfonate is irreversibly transferred (Liu et al., 2014). Thus, tetrathionate produced by tetrathionate reductase-type protein may be consumed by the reaction with TusA and DsrE in thiosulfate disproportionation in strain GF1^T^. In this case, sulfite derived from released thiosulfonate may eventually be passed to the Dsr system by sulfur relay, finally converted to sulfide according to the previously proposed mechanism (Santos et al., 2015; Figure 6). Furthermore, as recently proposed for *Desulfurivibrio alkaliphilus* (Thorup et al., 2017), it is possible that enzymes already known to be involved in sulfur metabolism have an unexpected function. Although further detailed biochemical or molecular genetic experiments are necessary to justify our hypothetical model reconstructed from the proteomic results, the model may represent a novel reaction pathway underlying thiosulfate disproportionation in strain GF1^T^.

Orthology analysis revealed that orthologous proteins of thiosulfate reductase-type protein were found in nearly half of the analyzed species (Supplementary Table 11). This may be partly because proteins orthologous to thiosulfate reductase-type protein can be possessed not only by thiosulfate-disproportionating bacteria but also by thiosulfate-reducing bacteria. Compared to thiosulfate reductase-type protein, orthologous proteins of tetrathionate reductase-type protein were less prevalent but present not only in strain GF1^T^ but also in 10 other sulfur-disproportionating species (Supplementary Table 11). Eight of these 10 species, with the exception of *Desulfobulbus propionicus* and *Caldimicrobium thiodismutans*, had orthologs of complete TtrBCA. An ortholog of tetrathionate reductase-type protein was also found in *Desulfatirhabdium butyrativorans*, which was reported to be unable to disproportionate thiosulfate and sulfite, but its inability should be confirmed using sulfide scavenger (Balk et al., 2008). These results indicated that tetrathionate reductase-type protein might be commonly possessed by many sulfur-disproportionating bacteria and associated with sulfur disproportionation. In addition, we found that bacteria with both an ortholog of tetrathionate reductase-type protein and orthologous proteins encoded by the YTD gene cluster tend to be able to perform sulfur disproportionation. Moreover, interestingly, most of them were derived from thermal environments, including deep-sea hydrothermal systems. Although the connection between the habitational and genetic features is unknown, these findings suggest that tetrathionate reductase-type protein, as well as proteins encoded by the YTD gene cluster, is one of the key proteins in sulfur disproportionation, especially for species inhabiting thermal environments.

Overall, the physiological and proteomic analyses of a novel sulfur-disproportionating *Desulfobulbaceae* species strain GF1^T^ provide a key insight into a unique involvement of tetrathionate reductase-type protein and proteins encoded by the YTD gene cluster in thiosulfate disproportionation. Furthermore, the identification of orthologs in this study will reinforce the hypothetical common metabolism of sulfur-disproportionating populations in thermal environments. Our findings provide a steppingstone toward elucidating the diversity of sulfur disproportionating bacteria and the genetic and metabolic evolution underlying sulfur disproportionation.

### Description of *Desulfolithobacter* gen. nov

*Desulfolithobacter* (De.sul.fo.li.tho.bac’ter. L. prep. *de*, from; L. neut. n. *sulfur*, sulfur; Gr. masc. n. *lithos*, stone; N.L. masc. n. *bacter*, rod; N.L. masc. n. *Desulfolithobacter*, a rod-shaped lithotrophic sulfate-reducer). Motile rods that stain Gram-negative. Strictly anaerobic. Mesophilic. Chemolithoautotrophic. Able to utilize molecular hydrogen as an electron donor and sulfate as an electron acceptor. NaCl is absolutely required for growth. The G + C content of genomic DNA is about 57%. Major cellular fatty acids are C_16:0_, C_18:1_, C_16:1_, and C_18:0_. Based on the 16S rRNA gene sequence, the genus *Desulfolithobacter* is distantly related to the genus *Desulfogranum*. Members of the genus *Desulfolithobacter* occur in deep-sea hydrothermal fields. The type species is *Desulfolithobacter dissulfuricans*.

### Description of *Desulfolithobacter dissulfuricans* sp. nov

*Desulfolithobacter dissulfuricans* (dis.sul. fu’ri.cans Gr. adv. *Dis*, in two; L. neut. n. *sulfur*, sulfur; N.L. part. Adj. *dissulfuricans*, disproportionating sulfur). Cells are motile, Gram-negative, short rods, and ~1.0–2.1 μm in length and 0.5–1.1 μm in width. Growth occurs between 25°C and 50°C, 20 and 40 g L^−1^ NaCl, and pH 6.1 and 6.8. Cells are strictly anaerobic. Chemolithoautotrophic growth occurs with H₂, formate, casamino acids, or malate as the electron donor and sulfate, sulfite, or thiosulfate as the electron acceptor. S^0^, thiosulfate, and tetrathionate are disproportionated. The major cellular fatty acids are C_16:0_, C_18:1_, C_16:1_, and C_18:0_. The major respiratory quinone is MK-8. The G + C DNA content is 56.9%. The type strain, GF1ᵀ (=JCM 34117^T^ = DSM 111414^T^), was isolated from the deep-sea hydrothermal vent chimney obtained from the Suiyo Seamount in the Izu-Bonin Arc, Japan.

## Data availability statement

The data presented in the study are deposited in the ProteomeXchange Consortium via the jPOST partner repository (https://jpostdb.org/), accession numbers JPST001840 and PXD036606 (Okuda et al., 2017), and in the GenBank/EMBL/DDBJ repository, accession numbers LC596831 and AP024233.

## Author contributions

YH, JM, KT, and SN designed this study. YH performed all the experiments except for the transmission electron microscope (TEM) observations and LC–MS/MS analysis. AT performed the TEM observations. YH and SSh prepared the samples for LC–MS/MS analysis. SSh conducted LC–MS/MS analysis. All authors contributed to the article and approved the submitted version.

## Funding

This work was partially supported by JSPS KAKENHI grant numbers JP 16H04843, 20H03322, and 19K05783.

## Conflict of interest

AT belongs to Depertment of Marine and Earth Sciences, Marine Works Japan Ltd.

The remaining authors declare that the research was conducted in the absence of any commercial or financial relationships that could be construed as a potential conflict of interest.

## Publisher’s note

All claims expressed in this article are solely those of the authors and do not necessarily represent those of their affiliated organizations, or those of the publisher, the editors and the reviewers. Any product that may be evaluated in this article, or claim that may be made by its manufacturer, is not guaranteed or endorsed by the publisher.

## Footnotes

^1^ https://services.birc.au.dk/hyddb/

^2^ http://enve-omics.ce.gatech.edu/aai/

## Abbreviations

ANI: average nucleotide identity
AAI: average amino acid identity
PSMs: peptide spectrum matches
NSAFs: normalized spectral abundance factors
TD: thiosulfate-disproportionating condition
SR: sulfate-reducing condition

## References

[ref1] Adam N. Han Y. Laufer-Meiser K. Bährle R. Schwarz-Schampera U. Schippers A.. (2021). Deltaproteobacterium strain KaireiS1, a mesophilic, hydrogen-oxidizing and sulfate-reducing bacterium from an inactive deep-sea hydrothermal chimney. Front. Microbiol. 12:686276. doi: 10.3389/fmicb.2021.686276, PMID: 34630341 PMC8494109

[ref2] Altschul S. F. Madden T. L. Schäffer A. A. Zhang J. Zhang Z. Miller W.. (1997). Gapped BLAST and PSI-BLAST: a new generation of protein database search programs. Nucleic Acids Res. 25, 3389–3402. doi: 10.1093/nar/25.17.3389, PMID: 9254694 PMC146917

[ref3] Aziz R. K. Bartels D. Best A. A. DeJongh M. Disz T. Edwards R. A.. (2008). The RAST server: rapid annotations using subsystems technology. BMC Genomics 9:75. doi: 10.1186/1471-2164-9-75, PMID: 18261238 PMC2265698

[ref4] Bak F. Cypionka H. (1987). A novel type of energy metabolism involving fermentation of inorganic Sulphur compounds. Nature 326, 891–892. doi: 10.1038/326891a0, PMID: 22468292

[ref5] Bak F. Pfennig N. (1987). Chemolithotrophic growth of *Desulfovibrio sulfodismutans* sp. nov. by disproportionation of inorganic sulfur compounds. Arch. Microbiol. 147, 184–189. doi: 10.1007/BF00415282

[ref6] Bak F. Schuhmann A. Jansen K.-H. (1993). Determination of tetrathionate and thiosulfate in natural samples and microbial cultures by a new, fast and sensitive ion chromatographic technique. FEMS Microbiol. Ecol. 12, 257–264. doi: 10.1111/j.1574-6941.1993.tb00038.x

[ref7] Balk M. Altinbaş M. Rijpstra W. I. C. Damsté J. S. S. Stams A. J. M. (2008). *Desulfatirhabdium butyrativorans* gen. nov., sp. nov., a butyrate-oxidizing, sulfate-reducing bacterium isolated from an anaerobic bioreactor. Int. J. Syst. Evol. Microbiol. 58, 110–115. doi: 10.1099/ijs.0.65396-0, PMID: 18175693

[ref8] Baross J. A. Deming J. W. (1995). “Growth at high temperatures: isolation and taxonomy, physiology, and ecology,” in The Microbiology of Deep-Sea Hydrothermal Vents. ed. Karl D. M. (Boca Raton: CRC Press), 169–217.

[ref9] Collins M. D. Widdel F. (1986). Respiratory Quinones of Sulphate-reducing and Sulphur-reducing bacteria: a systematic investigation. Syst. Appl. Microbiol. 8, 8–18. doi: 10.1016/S0723-2020(86)80141-2

[ref10] Dahl C. (2020). “Chapter 3 a biochemical view on the biological sulfur cycle,” in Environmental Technologies to Treat Sulphur Pollution: Principles and Engineering. ed. Lens P. N. L. (London, United Kingdom: IWA Publishing), 55–96.

[ref11] Dick G. J. (2019). The microbiomes of deep-sea hydrothermal vents: distributed globally, shaped locally. Nat. Rev. Microbiol. 17, 271–283. doi: 10.1038/s41579-019-0160-2, PMID: 30867583

[ref12] Duval S. Ducluzeau A. L. Nitschke W. Schoepp-Cothenet B. (2008). Enzyme phylogenies as markers for the oxidation state of the environment: the case of respiratory arsenate reductase and related enzymes. BMC Evol. Biol. 8:206. doi: 10.1186/1471-2148-8-206, PMID: 18631373 PMC2500031

[ref13] Elsgaard L. Guezennec J. Benbouzid-Rollet N. Prieur D. (1995). Mesophilic sulfate-reducing bacteria from three deep-sea hydrothermal vent sites. Oceanol. Acta 18, 95–104.

[ref14] Emms D. M. Kelly S. (2015). OrthoFinder: solving fundamental biases in whole genome comparisons dramatically improves orthogroup inference accuracy. Genome Biol. 16:157. doi: 10.1186/s13059-015-0721-2, PMID: 26243257 PMC4531804

[ref15] Emms D. M. Kelly S. (2019). OrthoFinder: phylogenetic orthology inference for comparative genomics. Genome Biol. 20:238. doi: 10.1186/s13059-019-1832-y, PMID: 31727128 PMC6857279

[ref16] Finster K. (2008). Microbiological disproportionation of inorganic sulfur compounds. J. Sulfur Chem. 29, 281–292. doi: 10.1080/17415990802105770

[ref17] Finster K. W. Kjeldsen K. U. Kube M. Reinhardt R. Mussmann M. Amann R.. (2013). Complete genome sequence of *Desulfocapsa sulfexigens*, a marine deltaproteobacterium specialized in disproportionating inorganic sulfur compounds. Stand. Genomic Sci. 8, 58–68. doi: 10.4056/sigs.3777412, PMID: 23961312 PMC3739170

[ref18] Finster K. Liesack W. Thamdrup B. (1998). Elemental sulfur and thiosulfate disproportionation by *Desulfocapsa sulfoexigens* sp. nov., a new anaerobic bacterium isolated from marine surface sediment. Appl. Environ. Microbiol. 64, 119–125. doi: 10.1128/AEM.64.1.119-125.1998, PMID: 9435068 PMC124681

[ref19] Florentino A. P. Pereira I. A. C. Boeren S. van den Born M. Stams A. J. M. Sánchez-Andrea I. (2019). Insight into the sulfur metabolism of *Desulfurella amilsii* by differential proteomics. Environ. Microbiol. 21, 209–225. doi: 10.1111/1462-2920.14442, PMID: 30307104 PMC6378623

[ref20] Fossing H. Jørgensen B. B. (1990). Oxidation and reduction of radiolabeled inorganic sulfur compounds in an estuarine sediment, Kysing Fjord, Denmark. Geochim. Cosmochim. Acta 54, 2731–2742. doi: 10.1016/0016-7037(90)90008-9

[ref21] Frederiksen T.-M. Finster K. (2003). Sulfite-oxido-reductase is involved in the oxidation of sulfite in *Desulfocapsa sulfoexigens* during disproportionation of thiosulfate and elemental sulfur. Biodegradation 14, 189–198. doi: 10.1023/A:1024255830925, PMID: 12889609

[ref22] Frederiksen T. M. Finster K. (2004). The transformation of inorganic sulfur compounds and the assimilation of organic and inorganic carbon by the sulfur disproportionating bacterium *Desulfocapsa sulfoexigens*. Antonie Van Leeuwenhoek 85, 141–149. doi: 10.1023/B:ANTO.0000020153.82679.f4, PMID: 15028874

[ref23] Friedrich M. Springer N. Ludwig W. Schink B. (1996). Phylogenetic positions of *Desulfofustis glycolicus* gen. nov., sp. nov., and *Syntrophobotulus glycolicus* gen. nov., sp. nov., two new strict anaerobes growing with glycolic acid. Int. J. Syst. Bacteriol. 46, 1065–1069. doi: 10.1099/00207713-46-4-1065, PMID: 8863436

[ref24] Frolova A. A. Slobodkina G. B. Baslerov R. V. Novikov A. A. Bonch-Osmolovskaya E. A. Slobodkin A. I. (2018). *Thermosulfurimonas marina* sp. nov., an autotrophic sulfur-disproportionating and nitrate-reducing bacterium isolated from a shallow-sea hydrothermal vent. Microbiology 87, 502–507. doi: 10.1134/S0026261718040082

[ref25] Galushko A. Kuever J. (2019). “Desulfovibrio” in Bergey’s Manual of Systematics of Archaea and Bacteria. eds. Trujillo M. E. Dedysh S. DeVos P. Hedlund B. Kämpfer P. Rainey F. A.. (Hoboken, New Jersey: John Wiley & Sons, Ltd).

[ref26] Galushko A. Kuever J. (2020). “*Desulfogranum* gen. nov” in Bergey’s Manual of Systematics of Archaea and Bacteria. eds. Trujillo M. E. Dedysh S. DeVos P. Hedlund B. Kämpfer P. Rainey F. A.. (Hoboken, New Jersey: John Wiley & Sons, Ltd).

[ref27] Gittel A. Kofoed M. V. W. Sørensen K. B. Ingvorsen K. Schramm A. (2012). Succession of *Deferribacteres* and *Epsilonproteobacteria* through a nitrate-treated high-temperature oil production facility. Syst. Appl. Microbiol. 35, 165–174. doi: 10.1016/j.syapm.2012.01.003, PMID: 22381470

[ref28] Goris J. Konstantinidis K. T. Klappenbach J. A. Coenye T. Vandamme P. Tiedje J. M. (2007). DNA-DNA hybridization values and their relationship to whole-genome sequence similarities. Int. J. Syst. Evol. Microbiol. 57, 81–91. doi: 10.1099/ijs.0.64483-0, PMID: 17220447

[ref29] Hashimoto Y. Tame A. Sawayama S. Miyazaki J. Takai K. Nakagawa S. (2021). *Desulfomarina profundi* gen. nov., sp. nov., a novel mesophilic, hydrogen-oxidizing, sulphate-reducing chemolithoautotroph isolated from a deep-sea hydrothermal vent chimney. Int. J. Syst. Evol. Microbiol. 71:005083. doi: 10.1099/ijsem.0.005083, PMID: 34739365

[ref30] Hensel M. Hinsley A. P. Nikolaus T. Sawers G. Berks B. C. (1999). The genetic basis of tetrathionate respiration in *salmonella typhimurium*. Mol. Microbiol. 32, 275–287. doi: 10.1046/j.1365-2958.1999.01345.x, PMID: 10231485

[ref31] Janssen P. H. Schuhmann A. Bak F. Liesack W. (1996). Disproportionation of inorganic sulfur compounds by the sulfate-reducing bacterium *Desulfocapsa thiozymogenes* gen. nov., sp. nov. Arch. Microbiol. 166, 184–192. doi: 10.1007/s002030050374

[ref32] Jørgensen B. B. (1990). A thiosulfate shunt in the sulfur cycle of marine sediments. Science 249, 152–154. doi: 10.1126/science.249.4965.152, PMID: 17836966

[ref33] Kawai S. Shimamura S. Shimane Y. Tsukatani Y. (2022). Proteomic time-course analysis of the filamentous anoxygenic phototrophic bacterium, *Chloroflexus aurantiacus*, during the transition from respiration to phototrophy. Microorganisms 10:1288. doi: 10.3390/microorganisms10071288, PMID: 35889008 PMC9316378

[ref34] Kelley D. S. Baross J. A. Delaney J. R. (2002). Volcanoes, fluids, and life at mid-ocean ridge spreading centers. Annu. Rev. Earth Planet. Sci. 30, 385–491. doi: 10.1146/annurev.earth.30.091201.141331

[ref35] Konstantinidis K. T. Rosselló-Móra R. Amann R. (2017). Uncultivated microbes in need of their own taxonomy. ISME J. 11, 2399–2406. doi: 10.1038/ismej.2017.113, PMID: 28731467 PMC5649169

[ref36] Kostka J. Nealson K. (1998). “Isolation, cultivation and characterization of iron-and manganese-reducing bacteria,” in Techniques in Microbial Ecology. eds. Burlage R. S. Atlas R. Stahl D. Geesey G. Sayler G. (Oxford, New York: Oxford University Press), 58–78.

[ref37] Krämer M. Cypionka H. (1989). Sulfate formation via ATP sulfurylase in thiosulfate-and sulfite-disproportionating bacteria. Arch. Microbiol. 151, 232–237. doi: 10.1007/BF00413135

[ref38] Kumar S. Stecher G. Li M. Knyaz C. Tamura K. (2018). MEGA X: molecular evolutionary genetics analysis across computing platforms. Mol. Biol. Evol. 35, 1547–1549. doi: 10.1093/molbev/msy096, PMID: 29722887 PMC5967553

[ref39] Lee I. Kim Y. O. Park S. C. Chun J. (2016). OrthoANI: an improved algorithm and software for calculating average nucleotide identity. Int. J. Syst. Evol. Microbiol. 66, 1100–1103. doi: 10.1099/ijsem.0.000760, PMID: 26585518

[ref40] Liu L. J. Stockdreher Y. Koch T. Sun S. T. Fan Z. Josten M.. (2014). Thiosulfate transfer mediated by DsrE/TusA homologs from acidothermophilic sulfur-oxidizing archaeon *Metallosphaera cuprina*. J. Biol. Chem. 289, 26949–26959. doi: 10.1074/jbc.M114.591669, PMID: 25122768 PMC4175335

[ref41] Lovley D. R. Phillips E. J. P. (1994). Novel processes for anaerobic sulfate production from elemental sulfur by sulfate-reducing bacteria. Appl. Environ. Microbiol. 60, 2394–2399. doi: 10.1128/aem.60.7.2394-2399.1994, PMID: 16349323 PMC201662

[ref42] Mardanov A. V. Beletsky A. V. Kadnikov V. V. Slobodkin A. I. Ravin N. V. (2016). Genome analysis of *Thermosulfurimonas dismutans*, the first thermophilic sulfur-disproportionating bacterium of the phylum *Thermodesulfobacteria*. Front. Microbiol. 7:950. doi: 10.3389/fmicb.2016.00950, PMID: 27379079 PMC4911364

[ref43] Muramatsu F. Tonomura M. Yamada M. Kasahara Y. Yamamura S. Iino T.. (2020). Possible involvement of a tetrathionate reductase homolog in dissimilatory arsenate reduction by *Anaeromyxobacter* sp. strain PSR-1. Appl. Environ. Microbiol. 86, e00829–e00820. doi: 10.1128/AEM.00829-20, PMID: 32978134 PMC7657633

[ref44] Nagata R. Takaki Y. Tame A. Nunoura T. Muto H. Mino S.. (2017). *Lebetimonas natsushimae* sp. nov., a novel strictly anaerobic, moderately thermophilic chemoautotroph isolated from a deep-sea hydrothermal vent polychaete nest in the mid-Okinawa trough. Syst. Appl. Microbiol. 40, 352–356. doi: 10.1016/j.syapm.2017.06.002, PMID: 28690052

[ref45] Nakagawa T. Nakagawa S. Inagaki F. Takai K. Horikoshi K. (2004). Phylogenetic diversity of sulfate-reducing prokaryotes in active deep-sea hydrothermal vent chimney structures. FEMS Microbiol. Lett. 232, 145–152. doi: 10.1016/S0378-1097(04)00044-8, PMID: 15033233

[ref46] Nakagawa S. Takai K. (2006). 3 the isolation of thermophiles from deep-sea hydrothermal environments. Methods Microbiol. 35, 55–91. doi: 10.1016/S0580-9517(08)70006-0

[ref47] Nakagawa S. Takai K. (2008). Deep-sea vent chemoautotrophs: diversity, biochemistry and ecological significance. FEMS Microbiol. Ecol. 65, 1–14. doi: 10.1111/j.1574-6941.2008.00502.x, PMID: 18503548

[ref48] Nakamura K. Takai K. (2014). Theoretical constraints of physical and chemical properties of hydrothermal fluids on variations in chemolithotrophic microbial communities in seafloor hydrothermal systems. Prog Earth Planet Sci 1:5. doi: 10.1186/2197-4284-1-5

[ref49] Okuda S. Watanabe Y. Moriya Y. Kawano S. Yamamoto T. Matsumoto M.. (2017). jPOSTrepo: an international standard data repository for proteomes. Nucleic Acids Res. 45, D1107–D1111. doi: 10.1093/nar/gkw1080, PMID: 27899654 PMC5210561

[ref50] Porter K. G. Feig Y. S. (1980). The use of DAPI for identifying and counting aquatic microflora. Limnol. Oceanogr. 25, 943–948. doi: 10.4319/lo.1980.25.5.0943

[ref51] Richter M. Rosselló-Móra R. (2009). Shifting the genomic gold standard for the prokaryotic species definition. Proc. Natl. Acad. Sci. U. S. A. 106, 19126–19131. doi: 10.1073/pnas.0906412106, PMID: 19855009 PMC2776425

[ref52] Rowe A. R. Chellamuthu P. Lam B. Okamoto A. Nealson K. H. (2015). Marine sediments microbes capable of electrode oxidation as a surrogate for lithotrophic insoluble substrate metabolism. Front. Microbiol. 5:784. doi: 10.3389/fmicb.2014.00784, PMID: 25642220 PMC4294203

[ref53] Sako Y. Takai K. Ishida Y. Uchida A. Katayama Y. (1996). *Rhodothermus obamensis* sp. nov., a modern lineage of extremely thermophilic marine bacteria. Int. J. Syst. Bacteriol. 46, 1099–1104. doi: 10.1099/00207713-46-4-1099, PMID: 8863442

[ref54] Santos A. A. Venceslau S. S. Grein F. Leavitt W. D. Dahl C. Johnston D. T.. (2015). A protein trisulfide couples dissimilatory sulfate reduction to energy conservation. Science 350, 1541–1545. doi: 10.1126/science.aad3558, PMID: 26680199

[ref55] Sass A. Rütters H. Cypionka H. Sass H. (2002). *Desulfobulbus mediterraneus* sp. nov., a sulfate-reducing bacterium growing on mono-and disaccharides. Arch. Microbiol. 177, 468–474. doi: 10.1007/s00203-002-0415-5, PMID: 12029392

[ref56] Sievert S. M. Hügler M. Taylor C. D. Wirsen C. O. (2008). “Sulfur oxidation at deep-sea hydrothermal vents,” in Microbial Sulfur Metabolism. eds. Dahl C. Friedrich C. G. (Berlin, Heidelberg: Springer), 238–258.

[ref57] Slobodkin A. I. Reysenbach A. L. Slobodkina G. B. Baslerov R. V. Kostrikina N. A. Wagner I. D.. (2012). *Thermosulfurimonas dismutans* gen. nov., sp. nov., an extremely thermophilic sulfur-disproportionating bacterium from a deep-sea hydrothermal vent. Int. J. Syst. Evol. Microbiol. 62, 2565–2571. doi: 10.1099/ijs.0.034397-0, PMID: 22199218

[ref58] Slobodkin A. I. Reysenbach A. L. Slobodkina G. B. Kolganova T. V. Kostrikina N. A. Bonch-Osmolovskaya E. A. (2013). *Dissulfuribacter thermophilus* gen. nov., sp. nov., a thermophilic, autotrophic, sulfur-disproportionating, deeply branching deltaproteobacterium from a deep-sea hydrothermal vent. Int. J. Syst. Evol. Microbiol. 63, 1967–1971. doi: 10.1099/ijs.0.046938-0, PMID: 23024145

[ref59] Slobodkin A. I. Slobodkina G. B. (2019). Diversity of sulfur-disproportionating microorganisms. Microbiology 88, 509–522. doi: 10.1134/S0026261719050138

[ref60] Slobodkin A. I. Slobodkina G. B. Panteleeva A. N. Chernyh N. A. Novikov A. A. Bonch-Osmolovskaya E. A. (2016). *Dissulfurimicrobium hydrothermale* gen. nov., sp. nov., a thermophilic, autotrophic, sulfur-disproportionating deltaproteobacterium isolated from a hydrothermal pond. Int. J. Syst. Evol. Microbiol. 66, 1022–1026. doi: 10.1099/ijsem.0.000828, PMID: 26646853

[ref61] Slobodkina G. B. Reysenbach A. L. Kolganova T. V. Novikov A. A. Bonch-Osmolovskaya E. A. Slobodkin A. I. (2017). *Thermosulfuriphilus ammonigenes* gen. nov., sp. nov., a thermophilic, chemolithoautotrophic bacterium capable of respiratory ammonification of nitrate with elemental sulfur. Int. J. Syst. Evol. Microbiol. 67, 3474–3479. doi: 10.1099/ijsem.0.002142, PMID: 28857038

[ref62] Søndergaard D. Pedersen C. N. S. Greening C. (2016). HydDB: a web tool for hydrogenase classification and analysis. Sci. Rep. 6:34212. doi: 10.1038/srep34212, PMID: 27670643 PMC5037454

[ref63] Stockdreher Y. Sturm M. Josten M. Sahl H. G. Dobler N. Zigann R.. (2014). New proteins involved in sulfur trafficking in the cytoplasm of *Allochromatium vinosum*. J. Biol. Chem. 289, 12390–12403. doi: 10.1074/jbc.M113.536425, PMID: 24648525 PMC4007435

[ref64] Stockdreher Y. Venceslau S. S. Josten M. Sahl H. G. Pereira I. A. C. Dahl C. (2012). Cytoplasmic sulfurtransferases in the purple sulfur bacterium *Allochromatium vinosum*: evidence for sulfur transfer from DsrEFH to DsrC. PLoS One 7:e40785. doi: 10.1371/journal.pone.0040785, PMID: 22815818 PMC3397948

[ref65] Tanabe T. S. Leimkühler S. Dahl C. (2019). “Chapter seven – the functional diversity of the prokaryotic sulfur carrier protein TusA,” in Advances in Microbial Physiology. ed. Poole R. K. (London, United Kingdom: Academic Press), 233–277. 10.1016/bs.ampbs.2019.07.004 31655739

[ref66] Tanizawa Y. Fujisawa T. Nakamura Y. (2018). DFAST: a flexible prokaryotic genome annotation pipeline for faster genome publication. Bioinformatics 34, 1037–1039. doi: 10.1093/bioinformatics/btx713, PMID: 29106469 PMC5860143

[ref67] Thorup C. Schramm A. Findlay A. J. Finster K. W. Schreiber L. (2017). Disguised as a sulfate reducer: growth of the deltaproteobacterium *Desulfurivibrio alkaliphilus* by sulfide oxidation with nitrate. MBio 8, e00671–e00617. doi: 10.1128/mBio.00671-17, PMID: 28720728 PMC5516251

[ref68] Umezawa K. Kojima H. Kato Y. Fukui M. (2020). Disproportionation of inorganic sulfur compounds by a novel autotrophic bacterium belonging to *Nitrospirota*. Syst. Appl. Microbiol. 43:126110. doi: 10.1016/j.syapm.2020.126110, PMID: 32847785

[ref69] Umezawa K. Kojima H. Kato Y. Fukui M. (2021). *Dissulfurispira thermophila* gen. nov., sp. nov., a thermophilic chemolithoautotroph growing by sulfur disproportionation, and proposal of novel taxa in the phylum *Nitrospirota* to reclassify the genus *Thermodesulfovibrio*. Syst. Appl. Microbiol. 44:126184. doi: 10.1016/j.syapm.2021.126184, PMID: 33676265

[ref70] Venceslau S. S. Stockdreher Y. Dahl C. Pereira I. A. C. (2014). The “bacterial heterodisulfide,” DsrC is a key protein in dissimilatory sulfur metabolism. Biochim. Biophys. Acta Bioenerg. 1837, 1148–1164. doi: 10.1016/j.bbabio.2014.03.007, PMID: 24662917

[ref71] Widdel F. Kohring G.-W. Mayer F. (1983). Studies on dissimilatory sulfate-reducing bacteria that decompose fatty acids III. Characterization of the filamentous gliding *Desulfonema limicola* gen. nov. sp. nov., and *Desulfonema magnum* sp. nov. Arch. Microbiol. 134, 286–294. doi: 10.1007/bf00407804

[ref72] Widdel F. Pfennig N. (1982). Studies on dissimilatory sulfate-reducing bacteria that decompose fatty acids II. Incomplete oxidation of propionate by *Desulfobulbus propionicus* gen. nov., sp. nov. Arch. Microbiol. 131, 360–365. doi: 10.1007/BF00411187 7283636

[ref73] Wu Y.-W. (2018). ezTree: an automated pipeline for identifying phylogenetic marker genes and inferring evolutionary relationships among uncultivated prokaryotic draft genomes. BMC Genomics 19:921. doi: 10.1186/s12864-017-4327-9, PMID: 29363425 PMC5780852

[ref74] Yarza P. Yilmaz P. Pruesse E. Glöckner F. O. Ludwig W. Schleifer K. H.. (2014). Uniting the classification of cultured and uncultured bacteria and archaea using 16S rRNA gene sequences. Nat. Rev. Microbiol. 12, 635–645. doi: 10.1038/nrmicro3330, PMID: 25118885

[ref75] Yu N. Y. Wagner J. R. Laird M. R. Melli G. Rey S. Lo R.. (2010). PSORTb 3.0: improved protein subcellular localization prediction with refined localization subcategories and predictive capabilities for all prokaryotes. Bioinformatics 26, 1608–1615. doi: 10.1093/bioinformatics/btq249, PMID: 20472543 PMC2887053

[ref76] Yvenou S. Allioux M. Slobodkin A. Slobodkina G. Jebbar M. Alain K. (2022). Genetic potential of *Dissulfurimicrobium hydrothermale*, an obligate sulfur-disproportionating thermophilic microorganism. Microorganisms 10:60. doi: 10.3390/microorganisms10010060, PMID: 35056509 PMC8780430

[ref77] Zeng X. Alain K. Shao Z. (2021). Microorganisms from deep-sea hydrothermal vents. Mar. Life Sci. Technol. 3, 204–230. doi: 10.1007/s42995-020-00086-4 PMC10077256 37073341

[ref78] Zybailov B. Mosley A. L. Sardiu M. E. Coleman M. K. Florens L. Washburn M. P. (2006). Statistical analysis of membrane proteome expression changes in *Saccharomyces cerevisiae*. J. Proteome Res. 5, 2339–2347. doi: 10.1021/pr060161n, PMID: 16944946

