# Supplementary material for: Physiological and comparative proteomic characterization of Desulfolithobacter dissulfuricans gen. nov., sp. nov., a novel mesophilic, sulfur-disproportionating chemolithoautotroph from a deep-sea hydrothermal vent
Source: Front Microbiol. 2022 Dec 1;13:1042116. doi: 10.3389/fmicb.2022.1042116 (PMC9751629; doi:10.3389/fmicb.2022.1042116)
Supplement: Supplementary file 2 [file Data_Sheet_1.DOCX]

Supplementary Material

# Supplementary Tables

Supplementary Table 1. List of single-copy marker genes used for the reconstruction of the　phylogenomic tree shown in Figure 2B.

| **Pfam accession** | **Pfam summary** |
| --- | --- |
| PF13507.5 | GATase_5, CobB/CobQ-like |
| PF00203.20 | Ribosomal_S19, Ribosomal |
| PF03772.15 | Competence, Competence |
| PF03819.16 | MazG, MazG |
| PF02527.14 | GidB, rRNA |
| PF06418.13 | CTP_synth_N, CTP |
| PF00410.18 | Ribosomal_S8, Ribosomal |
| PF12002.7 | MgsA_C, MgsA |
| PF03968.13 | OstA, OstA-like |
| PF02601.14 | Exonuc_VII_L, Exonuclease |
| PF00398.19 | RrnaAD, Ribosomal |
| PF09334.10 | tRNA-synt_1g, tRNA |
| PF01121.19 | CoaE, Dephospho-CoA |
| PF00213.17 | OSCP, ATP |
| PF01165.19 | Ribosomal_S21, Ribosomal |
| PF01195.18 | Pept_tRNA_hydro, Peptidyl-tRNA |
| PF00453.17 | Ribosomal_L20, Ribosomal |
| PF01502.17 | PRA-CH, Phosphoribosyl-AMP |
| PF01746.20 | tRNA_m1G_MT, tRNA |
| PF02684.14 | LpxB, Lipid-A-disaccharide |
| PF04127.14 | DFP, DNA |
| PF00687.20 | Ribosomal_L1, Ribosomal |
| PF00252.17 | Ribosomal_L16, Ribosomal |
| PF00290.19 | Trp_syntA, Tryptophan |
| PF02569.14 | Pantoate_ligase, Pantoate-beta-alanine |
| PF00237.18 | Ribosomal_L22, Ribosomal |
| PF02325.16 | YGGT, YGGT |
| PF00488.20 | MutS_V, MutS |
| PF01795.18 | Methyltransf_5, MraW |
| PF02576.16 | DUF150, RimP |
| PF00162.18 | PGK, Phosphoglycerate |
| PF00861.21 | Ribosomal_L18p, Ribosomal |
| PF01118.23 | Semialdhyde_dh, Semialdehyde |
| PF02016.14 | Peptidase_S66, LD-carboxypeptidase |
| PF14450.5 | FtsA, Cell |
| PF00677.16 | Lum_binding, Lumazine |
| PF03840.13 | SecG, Preprotein |
| PF04997.11 | RNA_pol_Rpb1_1, RNA |
| PF01170.17 | UPF0020, Putative |
| PF00312.21 | Ribosomal_S15, Ribosomal |
| PF13525.5 | YfiO, Outer |
| PF02618.15 | YceG, YceG-like |
| PF00829.20 | Ribosomal_L21p, Ribosomal |
| PF04551.13 | GcpE, GcpE |
| PF00318.19 | Ribosomal_S2, Ribosomal |
| PF00297.21 | Ribosomal_L3, Ribosomal |
| PF00183.17 | HSP90, Hsp90 |
| PF00885.18 | DMRL_synthase, 6,7-dimethyl-8-ribityllumazine |
| PF00338.21 | Ribosomal_S10, Ribosomal |
| PF01634.17 | HisG, ATP |
| PF01782.17 | RimM, RimM |
| PF01327.20 | Pep_deformylase, Polypeptide |
| PF04453.13 | OstA_C, Organic |
| PF01967.20 | MoaC, MoaC |
| PF02602.14 | HEM4, Uroporphyrinogen-III |
| PF01084.19 | Ribosomal_S18, Ribosomal |
| PF00698.20 | Acyl_transf_1, Acyl |
| PF00380.18 | Ribosomal_S9, Ribosomal |
| PF01016.18 | Ribosomal_L27, Ribosomal |
| PF03719.14 | Ribosomal_S5_C, Ribosomal |
| PF14622.5 | Ribonucleas_3_3, Ribonuclease-III-like |
| PF00177.20 | Ribosomal_S7, Ribosomal |
| PF03588.13 | Leu_Phe_trans, Leucyl/phenylalanyl-tRNA |
| PF02381.17 | MraZ, MraZ |
| PF01784.17 | NIF3, NIF3 |
| PF00154.20 | RecA, recA |
| PF07479.13 | NAD_Gly3P_dh_C, NAD-dependent |
| PF03947.17 | Ribosomal_L2_C, Ribosomal |
| PF00709.20 | Adenylsucc_synt, Adenylosuccinate |
| PF08497.9 | Radical_SAM_N, Radical |
| PF00342.18 | PGI, Phosphoglucose |
| PF00490.20 | ALAD, Delta-aminolevulinic |
| PF00347.22 | Ribosomal_L6, Ribosomal |
| PF02699.14 | YajC, Preprotein |
| PF12631.6 | MnmE_helical, MnmE |
| PF01765.18 | RRF, Ribosome |
| PF06574.11 | FAD_syn, FAD |
| PF02617.16 | ClpS, ATP-dependent |
| PF02367.16 | TsaE, Threonylcarbamoyl |
| PF01245.19 | Ribosomal_L19, Ribosomal |
| PF02594.15 | DUF167, Uncharacterised |
| PF02882.18 | THF_DHG_CYH_C, Tetrahydrofolate |
| PF03477.15 | ATP-cone, ATP |
| PF02033.17 | RBFA, Ribosome-binding |
| PF04286.11 | DUF445, Protein |
| PF03602.14 | Cons_hypoth95, Conserved |
| PF01288.19 | HPPK, 7,8-dihydro-6-hydroxymethylpterin-pyrophosphokinase |
| PF00562.27 | RNA_pol_Rpb2_6, RNA |
| PF02224.17 | Cytidylate_kin, Cytidylate |
| PF01176.18 | eIF-1a, Translation |
| PF03946.13 | Ribosomal_L11_N, Ribosomal |
| PF01678.18 | DAP_epimerase, Diaminopimelate |
| PF01029.17 | NusB, NusB |
| PF01649.17 | Ribosomal_S20p, Ribosomal |
| PF00475.17 | IGPD, Imidazoleglycerol-phosphate |
| PF00466.19 | Ribosomal_L10, Ribosomal |
| PF00673.20 | Ribosomal_L5_C, ribosomal |
| PF04257.13 | Exonuc_V_gamma, Exodeoxyribonuclease |
| PF02575.15 | YbaB_DNA_bd, YbaB/EbfC |
| PF02410.14 | RsfS, Ribosomal |
| PF01668.17 | SmpB, SmpB |
| PF14748.5 | P5CR_dimer, Pyrroline-5-carboxylate |
| PF02401.17 | LYTB, LytB |
| PF01018.21 | GTP1_OBG, GTP1/OBG |
| PF03749.12 | SfsA, Sugar |
| PF02223.16 | Thymidylate_kin, Thymidylate |
| PF01071.18 | GARS_A, Phosphoribosylglycinamide |
| PF00464.18 | SHMT, Serine |
| PF00091.24 | Tubulin, Tubulin/FtsZ |
| PF00889.18 | EF_TS, Elongation |
| PF04085.13 | MreC, rod |
| PF01933.17 | UPF0052, Uncharacterised |
| PF11987.7 | IF-2, Translation-initiation |
| PF13292.5 | DXP_synthase_N, 1-deoxy-D-xylulose-5-phosphate |
| PF00828.18 | Ribosomal_L27A, Ribosomal |
| PF01379.19 | Porphobil_deam, Porphobilinogen |
| PF01509.17 | TruB_N, TruB |
| PF05173.13 | DapB_C, Dihydrodipicolinate |
| PF02673.17 | BacA, Bacitracin |
| PF11898.7 | DUF3418, Domain |
| PF02091.14 | tRNA-synt_2e, Glycyl-tRNA |
| PF03205.13 | MobB, Molybdopterin |
| PF02547.14 | Queuosine_synth, Queuosine |
| PF01411.18 | tRNA-synt_2c, tRNA |
| PF02609.15 | Exonuc_VII_S, Exonuclease |
| PF00344.19 | SecY, SecY |
| PF07497.11 | Rho_RNA_bind, Rho |
| PF00572.17 | Ribosomal_L13, Ribosomal |
| PF00227.25 | Proteasome, Proteasome |
| PF01196.18 | Ribosomal_L17, Ribosomal |
| PF00542.18 | Ribosomal_L12, Ribosomal |
| PF01255.18 | Prenyltransf, Putative |
| PF02578.14 | Cu-oxidase_4, Multi-copper |
| PF04552.12 | Sigma54_DBD, Sigma-54, |
| PF00327.19 | Ribosomal_L30, Ribosomal |
| PF13393.5 | tRNA-synt_His, Histidyl-tRNA |
| PF01487.14 | DHquinase_I, Type |
| PF02606.13 | LpxK, Tetraacyldisaccharide-1-P |
| PF02092.16 | tRNA_synt_2f, Glycyl-tRNA |
| PF02502.17 | LacAB_rpiB, Ribose/Galactose |
| PF02620.16 | DUF177, Uncharacterized |
| PF03054.15 | tRNA_Me_trans, tRNA |
| PF01346.17 | FKBP_N, Domain |
| PF00625.20 | Guanylate_kin, Guanylate |
| PF08459.10 | UvrC_HhH_N, UvrC |

Supplementary Table 2. Atom percentage of ^15^N in total N (^15^N atom%) in strain GF1^T^ cells cultured with N_2_ as the sole nitrogen source. nd, not detected.

|  | **^15^N atom% of microbial biomass** | | |
| --- | --- | --- | --- |
| **Culture condition** | **^15^N_2_ culture** | **^15^N_2_-free culture** | **^15^N_2_-free uninoculated medium** |
| **Sulfate reduction** | 0.71 | 0.37 | nd |
| **S^0^ disproportionation** | 0.77 | 0.37 | nd |
| **Thiosulfate disproportionation** | 1.04 | 0.37 | nd |

**Supplementary Table 3.** List of genes involved in sulfur, carbon, and nitrogen metabolism.

| **Metabolism** | **Gene name** | **Locus tag** |
| --- | --- | --- |
| Sulfur metabolism | *sat* | GF1_19970 |
|  | *aprA* | GF1_23660 |
|  | *aprB* | GF1_23670 |
|  | *dsrA* | GF1_25910 |
|  | *dsrB* | GF1_25900 |
|  | *dsrC* | GF1_24490 |
|  | *dsrD* | GF1_25890 |
|  | *dsrM* | GF1_26980 |
|  | *dsrK* | GF1_26970 |
|  | *dsrJ* | GF1_26960 |
|  | *dsrO* | GF1_26950 |
|  | *dsrP* | GF1_26940 |
|  | *qmoA* | GF1_23650 |
|  | *qmoB* | GF1_23640 |
|  | *qmoC* | GF1_23630 |
| Carbon metabolism (Carbon fixation) | *fdhA* | GF1_14240 |
|  | *fhs* | GF1_21850 |
|  | *folD* | GF1_14180 |
|  | *metF, acsD* | GF1_14200 |
|  | *acsA* | GF1_01790, GF1_14190 |
|  | *acsB* | GF1_14210 |
|  | *acsC* | GF1_14220 |
|  | *acsE* | GF1_31690 |
| Nitrogen metabolism (Nitrogen fixation) | *nifH* | GF1_02420 |
|  | *nifD* | GF1_02390 |
|  | *nifK* | GF1_02380 |
|  | *nifE* | GF1_02290 |
|  | *nifN* | GF1_02280 |
|  | *nifB* | GF1_02270, GF1_02370 |

**Supplementary Table 4-14 are provided as a separate spreadsheet file.**

# Supplementary Figures


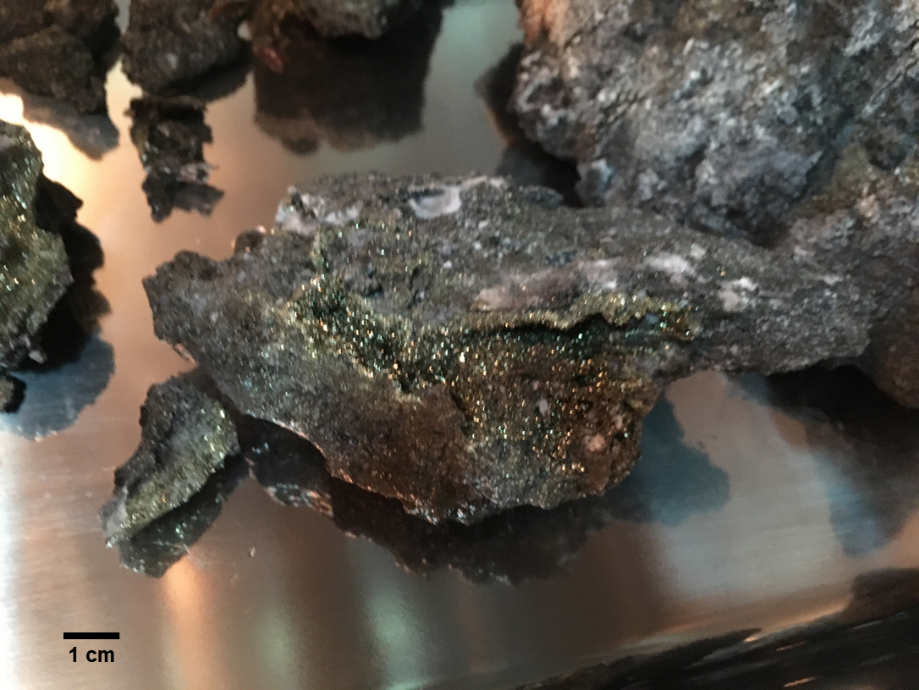


Supplementary Figure 1. The chimney structure obtained from a deep-sea hydrothermal vent at the Suiyo Seamount.


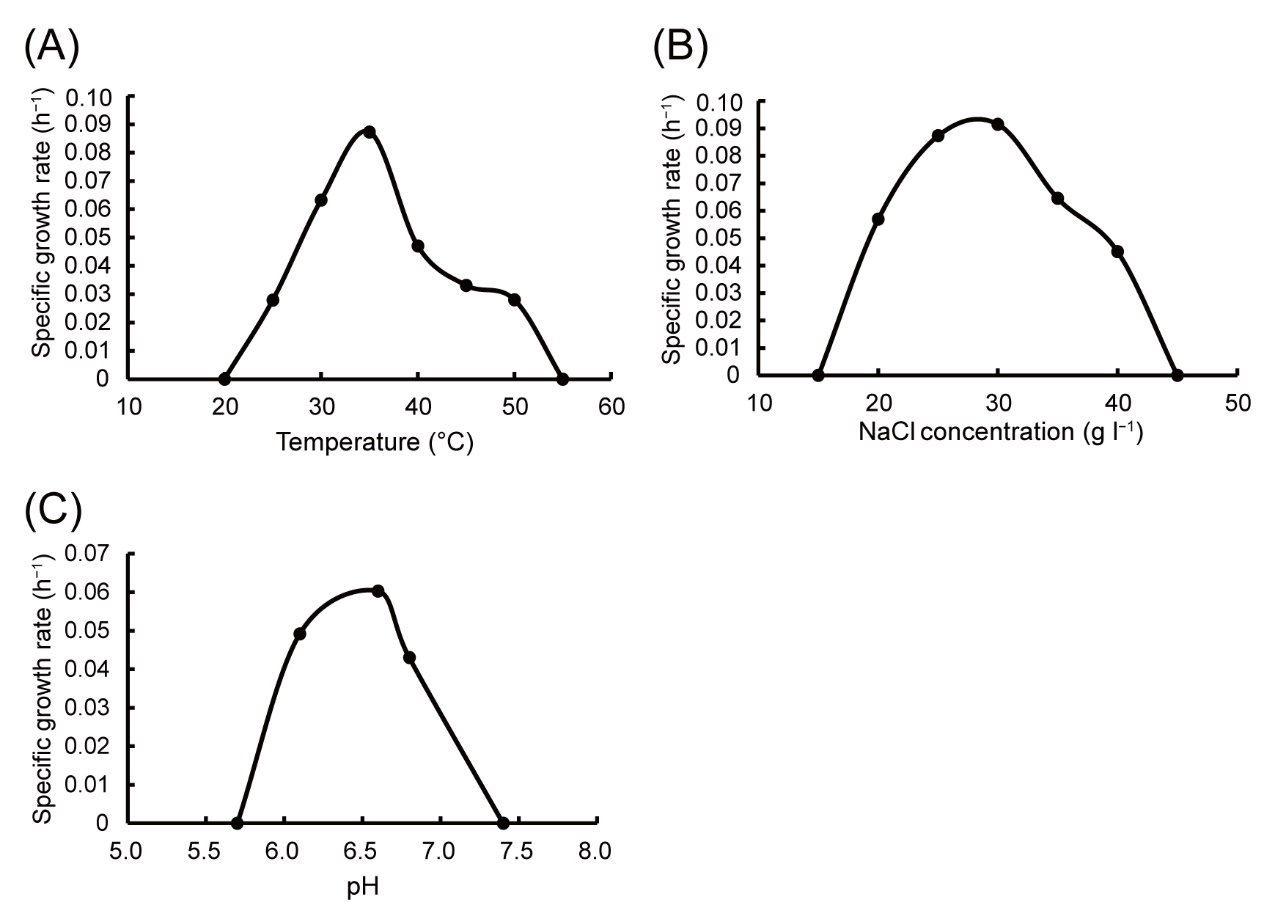


Supplementary Figure 2. Effects of temperature **(A)**, NaCl concentration **(B)**, and pH **(C)** on the growth of strain GF1^T^. Growth curves at different temperatures were determined in MMJFE medium. The effect of NaCl concentration on growth was determined in MMJFE medium with various concentration of NaCl. The effect of pH on growth was determined in MMJFE medium at 35°C, over a range of pH values. The pH was adjusted at room temperature.


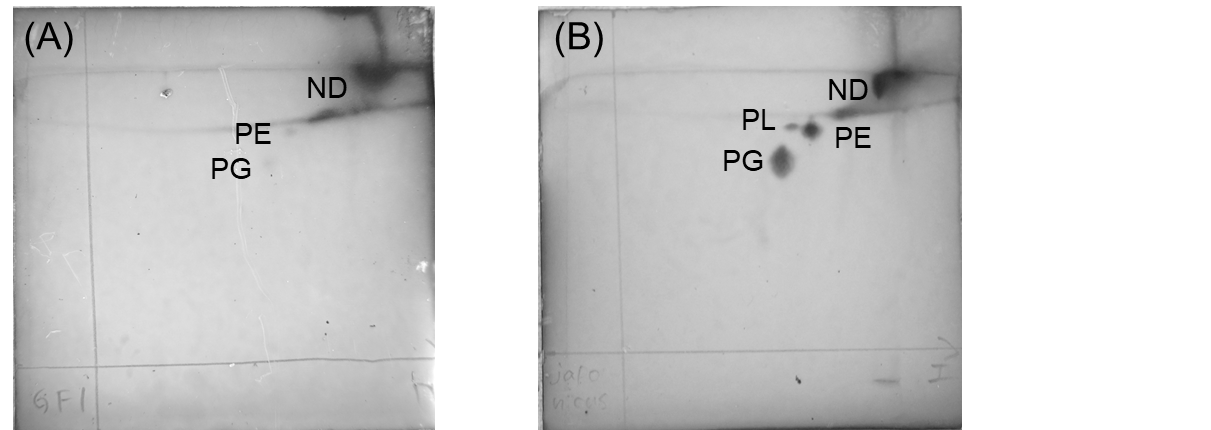
Supplementary Figure 3. Polar lipids of strain GF1^T^ **(A)** and *Desulfogranum japonicum* Pro1^T^ **(B)**. PE, phosphatidylethanolamine; PG, phosphatidylglycerol; PL, unidentified phospholipid; ND, not determined.

**
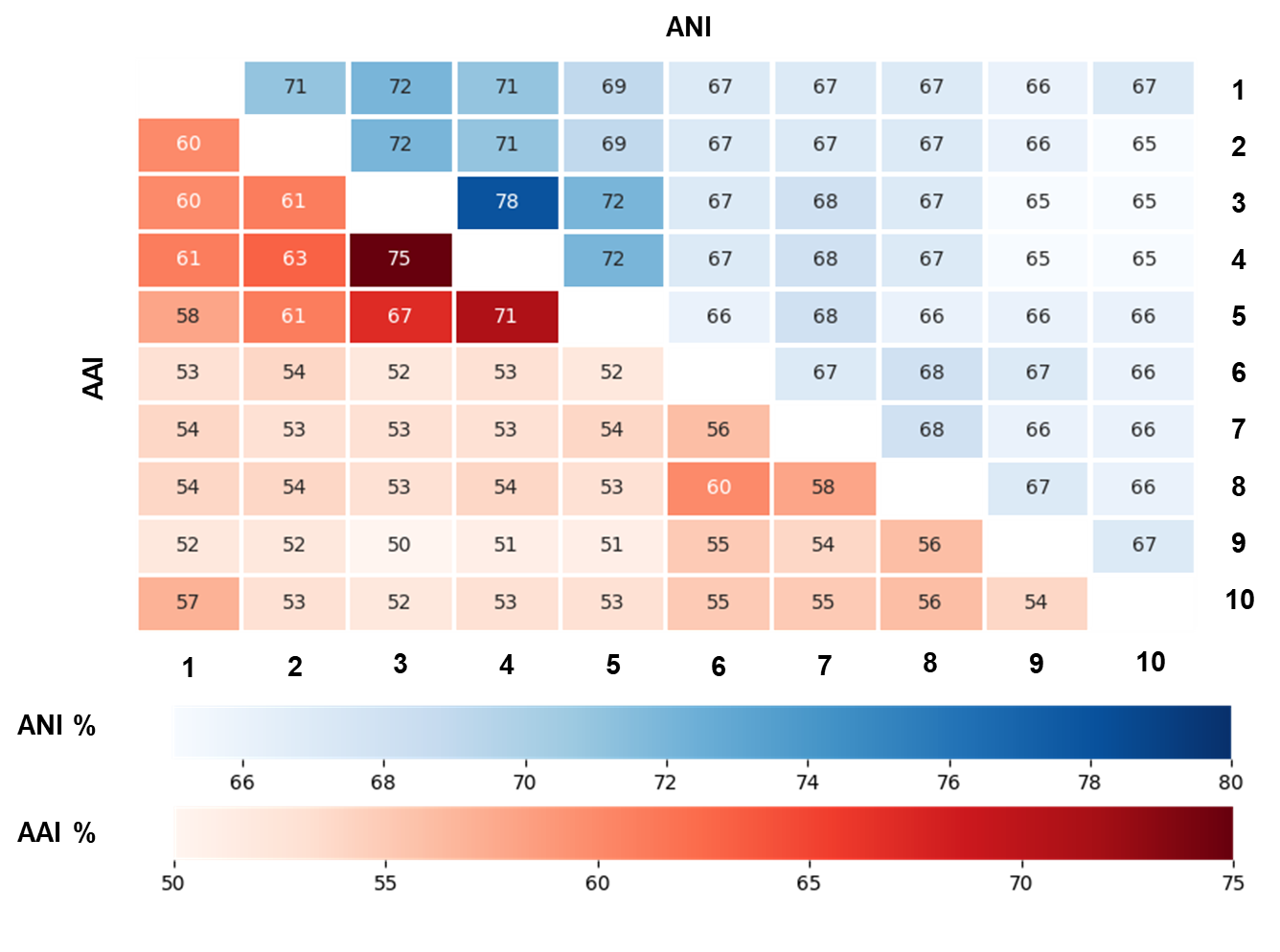
**

Supplementary Figure 4. ANI and AAI values between strain GF1^T^ and related genera of *Desulfobulbaceae*. 1, GF1^T^ (this study) (AP024233); 2, *Desulfogranum mediterraneum* 86FS1^T^ (NZ_AUCW00000000); 3, *Desulfobulbus elongatus* FP^T^ (NZ_JHZB00000000); 4, *Desulfobulbus propionicus* 1pr3^T^ (CP002364); 5, *Desulfobulbus rhabdoformis* M16^T^ (NZ_JAFFQA000000000); 6, *Desulforhopalus singaporensis* S'pore T1^T^ (NZ_FNJI00000000); 7, *Desulfofustis glycolicus* PerGlyS^T^ (NZ_FQXS00000000); 8, *Desulfopila aestuarii* MSL86^T^ (NZ_FRFE00000000); 9, *Desulfotalea psychrophila* LSv54^T^ (CR522870); 10, *Desulfocapsa sulfexigens* SB164P1^T^ (CP003985).


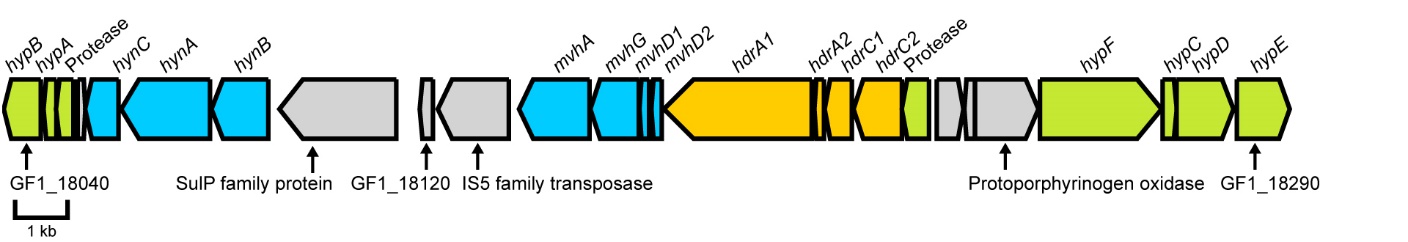


Supplementary Figure 5. Gene arrangement of hydrogenase gene cluster in the genome of strain GF1^T^. Green, hydrogenase accessory protein; Blue, hydrogenase; Orange, heterodisulfide reductase; Gray, hypothetical proteins or proteins not related to hydrogen metabolism. Abbreviations: Protease, hydrogenase maturation protease.
